# Intrinsic timescales of sensory integration for motion perception

**DOI:** 10.1038/s41598-019-40649-9

**Published:** 2019-03-08

**Authors:** Woochul Choi, Se-Bum Paik

**Affiliations:** 1 Department of Bio and Brain Engineering, Korea Advanced Institute of Science and Technology, Daejeon, 34141 Republic of Korea; 2 Program of Brain and Cognitive Engineering, Korea Advanced Institute of Science and Technology, Daejeon, 34141 Republic of Korea

## Abstract

A subject-specific process of perceptual decision making is of importance to how the brain translates its interpretation of sensory information into behavior. In particular, a number of studies reported substantial variation across the observers’ decision behavior, which may reflect different profiles of evidence accumulated by each individual. However, a detailed profile of perceptual integration has not yet been verified from human behavioral data. To address the issue, we precisely measured the time course of sensory integration, as the “sensory integration kernel” of subjects, using a coherence-varying motion discrimination task. We found that each subject has a distinct profile of sensory integration. We observed that kernel size (maximum sensory integration interval) is consistent within subjects, independent of external stimuli conditions. The observed kernel could accurately predict subject-specific perceptual behaviors and explain the inter-individual variation of observed behaviors. Surprisingly, the performance of most subjects did not improve in proportion to increased duration of the stimulus, but was maximized when the stimulus duration matched their kernel size. We also found that the observed kernel size was strongly correlated with the subject-specific perceptual characteristics for illusory motion. Our results suggest that perceptual decisions arise from intrinsic decision dynamics, and on individual timescales of sensory integration.

## Introduction

Perceptual decision making is the act of choosing an option based on the evaluation of sensory evidence^1^. To understand how the brain translates the interpretation of sensory information into behavior, it is essential to study the mechanism by which this psychophysical judgment process occurs^2–4^. To address this issue, human behavior in visual tasks such as motion detection has been studied extensively^2,5,6^. In such studies, a net motion direction discrimination task has been frequently implemented with a dynamic random dot display and observers’ response characteristics (i.e., reaction time, accuracy, decision confidence) were measured^2,7–11^. Thereafter, neurophysiological studies examined the relationship between neural activity patterns and psychophysical behavior in monkeys, revealing a strong correlation between the neuronal and behavioral data^2,5,7,12^. Similarly, computational models suggested that perceptual decision making arises through the integration of sensory information^8,10,11^ and can be described by the diffusion-to-boundary process model^9,13,14^.

In a number of studies of visual perception, however, human behavioral data indicate a substantial variation across the observer behavior even when an identical stimulus is given^1,11,15,16^. This inter-individual variability in perceptual behavior, often ignored or considered noise, has been recently studied more carefully using brain imaging techniques and individual variability appears to be related to local structure or connectivity of the brain^17,18^. Further research is required, as the notion that inter-individual differences in perceptual decisions should be considered structural variations of neural circuits as opposed to mere statistical noise remains under debate.

A recent study on the perceptual decision making process during a motion perception task^11^ suggested that subjective decision times reflects different profiles of evidence accumulated by each individual and showed that the bounded evidence accumulation model^13,14^ could predict subject behavior from their observed decision time. This suggests that inter-individual variability in perceptual decision time may be due to the diverse computation of the decision variable and the wide variation of decision threshold in individuals, and may be of particular importance for those investigating the origin of inter-individual variability in perceptual behavior.

Given this, we hypothesized that if perceptual decisions reflect individual characteristics of each brain circuit, then the individuals have a unique sensory integration profile to make a perceptual decision. Specifically, we assumed that the time course of sensory integration needed to make a single perceptual decision– termed a “sensory integration kernel” – will be consistent within a subject, independent of instantaneous stimulus dynamics. We anticipate that this intrinsic kernel size may vary across subjects and this may be an origin of inter-individual variability in perceptual behavior. Therefore, we suggest that wide variation in perceptual behavior originates from the intrinsic characteristics of brain circuits of individuals for sensory integration and that this should be considered as crucial information of subject-specific characteristics of perception.

To validate our hypothesis, we performed a series of psychophysics experiments using a random dot display which motion coherence temporally varies – coherence-varying motion discrimination task. We measured a temporal sensory integration kernel of each individual by estimating the motion coherence pattern that triggered perceptual decision, using stimuli of various temporal dynamics. We observed that each subject has a very consistent length and profile of temporal kernel, independent of the stimulus dynamics given. Observed kernel size (the maximum integration interval) largely varied across subjects and accurately predicted the inter-individual variability in responses. Additionally, we found that the kernel size-matched motion stimulus maximized the probability of correct response in each individual performance. Furthermore, we found that subjects’ characteristics of illusory motion perception were highly correlated with the observed intrinsic kernel. Therefore, our results suggest that an intrinsic, perceptual kernel is a critical factor to study sensory perception and that the inter-individual variability can be considered as a subject-specific trait from this sensory integration kernel.

## Results

### Perceptual decision making during coherence-varying motion discrimination task

**Figure 1:**
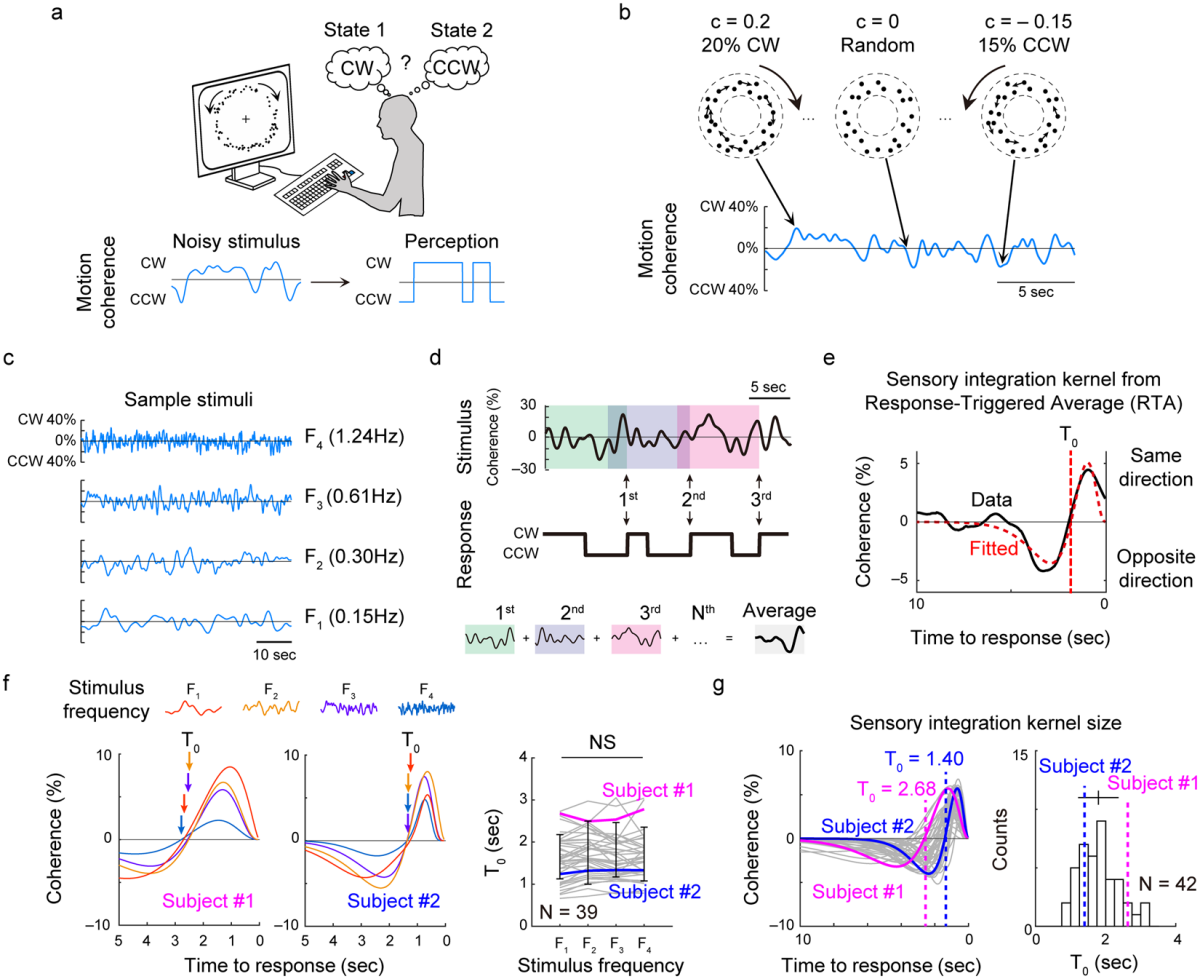
Measurement of evidence accumulation time course using coherence-varying motion discrimination task. (**a**) Dots positioned at random locations in a circular annulus were given as a visual stimulus. Subjects were asked to report the direction of perceived rotational motion by keyboard press. The positions of dots were updated at every 50 ms and the perceptual alternations between the two directions were recorded. (**b**) A portion (motion coherence, **c**) of dots were controlled to rotate either clockwise or counter-clockwise, and the motion coherence was designed to change over time (**c**) 60 second-long motion coherence pattern was designed to fluctuate with four different temporal frequencies, from 0.15 Hz (F_1_)~1.24 Hz (F_4_). (**d**) At each response of motion perception (i.e. black arrows for CW switches), the preceding motion coherence pattern was recorded and averaged. (**e**) From the observed Response-Triggered Average (RTA), the time point at which the curve becomes zero was defined as T_0_, the positive sensory integration time interval. (**f**) Sensory integration kernel under four different stimulus conditions. T_0_ was not significantly different under four conditions (repeated measure ANOVA, p = 0.11, F(3, 114) = 2.02, N = 39). (**g**) Fitted sensory integration kernel of all subjects. Two sample kernels were highlighted for comparison. Subject 1 (magenta) showed a longer kernel of T_0_ = 2.68 sec than subject 2 (blue) with a kernel of T_0_ = 1.40 sec. T_0_ varied from approximately 1–3.5 sec across subjects (N = 42).

To characterize individual motion perception sensory integration, we designed a coherence-varying motion discrimination task with random dot display. For a motion stimulus, black dots were positioned in a circular annulus and a certain portion of the dots in each frame was shifted to new rotated positions (clockwise or counter-clockwise) in the next movie frame, while other dots were randomly positioned. During a single 60 s trial, the ratio of rotating dots (motion coherence, c) and a direction of rotation (sign of c) change over time (Fig. 1a,b, see the Methods section for details, see also ref.^10^). Because we are interested in how instantaneous fluctuating motion dynamics drive the perceptual decision, we designed the motion coherence pattern so it can fluctuate at four different frequencies. By applying Gaussian filters of four different widths to the random number, the 60 s coherence pattern can fluctuate with central frequency varying from 0.15 Hz (F_1_; lowest) to 1.24 Hz (F_4_; highest), which was fixed for a single trial (Fig. 1c, see details in Method, Supplementary Fig. S1, Movies S1–S4). The estimated motion energy in the angular direction^10,19–21^ confirmed that the designed motion coherence pattern is well embedded in the random dot stimulus. This motion coherence pattern was used to represent the global rotational motion in further analysis (Supplementary Fig. S2, see details in Method). During the observation, subjects were asked to report the perceived direction of global rotation – clockwise (CW), counter-clockwise (CCW) or ambiguous—as soon as they perceived a rotational motion. So, the subjects reported the direction of rotation whenever their perceived direction was switched from the previously perceived rotational direction (e.g., report CCW when perceived direction switches from CW to CCW). As a result, individual perceptual response patterns to a given motion coherence pattern were obtained (Fig. 1d, middle).

To quantify the subject’s perceptual sensory integration kernel, we measured the average motion coherence pattern that triggered perceptual responses using the reverse correlation method^22–24^. We captured the motion coherence pattern within the 10-second window prior to the time point whenever subject reported the direction of the perceived motion (Fig. 1d). Then, the sampled motion coherence patterns were averaged together, creating the response-triggered average stimulus (RTA). The RTA measured in each subject allowed us to find the temporal profile of sensory integration for a perceptual decision, which we defined as the “sensory integration kernel” of the subject (Fig. 1e). Temporal profile of the RTA showed two windows of opposite sign: stimulus right before response drives decision to the same (positive) direction with a given motion, and stimulus far before drives decision in the opposite (negative) direction (see Supplementary Fig. S3 for control analysis). We found that an individual RTA curve fit well to a superposition of two alpha functions, similar to the quantification of the temporal receptive field structure of retinal neurons^25^.

1 $$RTA(t)={A}_{1}{(\frac{t}{{\tau }_{1}})}^{n}{e}^{-\frac{(n-1)t}{{\tau }_{1}}}-{A}_{2}{(\frac{t}{{\tau }_{2}})}^{n}{e}^{-\frac{(n-1)t}{{\tau }_{2}}}$$

We focused on the parameter T_0_, i.e. the timing that the RTA first crosses the zero-coherence (temporal window of positive sensory integration), for the profile of this kernel because this value reveals the size of the temporal window for effective sensory integration for decision making. Another parameter, A_pos_, i.e., the positive amplitude of the RTA, reveals how strong the instantaneous motion signal at time t must be on average to induce a perceptual decision, which also reveals the individual characteristics of sensory integration along with T_0_. Thus, integration of the RTA amplitude, or the area, illustrates how much a visual motion signal can induce a perceptual decision in each individual. Although four parameters are required to describe a complete kernel profile (e.g., amplitude of positive/negative peaks and width of positive/negative windows), we assumed that T_0_ can represent the individual characteristics of the kernel in this study, which directly indicates the maximum size of the sensory integration window (see Supplementary Fig. S4 for detailed shape parameters and their characteristics).

We first compared the observed RTA curves across different stimulus dynamics conditions and found that T_0_ values (the positive kernel sizes) were consistent across stimulus conditions, even though the frequency of motion fluctuation changed 8-fold (Fig. 1f). We confirmed that the differences in T_0_ across the stimulus conditions were insignificant in our sample (p = 0.11, F(3, 114) = 2.02, repeated measure ANOVA, Bayes factor = 0.054, N = 39, T_0_ = 1.71 ± 0.64, 1.82 ± 0.65, 1.75 ± 0.75, and 1.65 ± 0.53 for F_1_, F_2_, F_3_, and F_4_, respectively, mean ± S.D). This suggests that the time course of motion integration within an individual is fairly consistent and independent of the stimulus dynamics. We then averaged the RTAs from all four conditions to obtain an average sensory integration kernel for each subject. In the averaged RTA – sensory integration kernel, we found that the kernel size T_0_ varied noticeably from 0.8 to 3.5 sec across individuals (Fig. 1g). We also confirmed this result from the analysis of local motion energy of actual stimuli presented to the subjects. The temporal profile of the motion energy kernel in the local spatial segments was not significantly different from that obtained from the global motion coherence pattern (repeated-measures ANOVA, F(4, 160) = 1.66, p = 0.16, N = 41, Bayes factor = 0.032), which confirms that the observed kernel is an intrinsic characteristic of each subject (See Supplementary Fig. S2). Additionally, we also observed that the intrinsic profile of the kernel is maintained even when there exists an imbalance between the CW and CCW decisions or inter-decision-intervals (See Supplementary Fig. S5).

**Figure 2:**
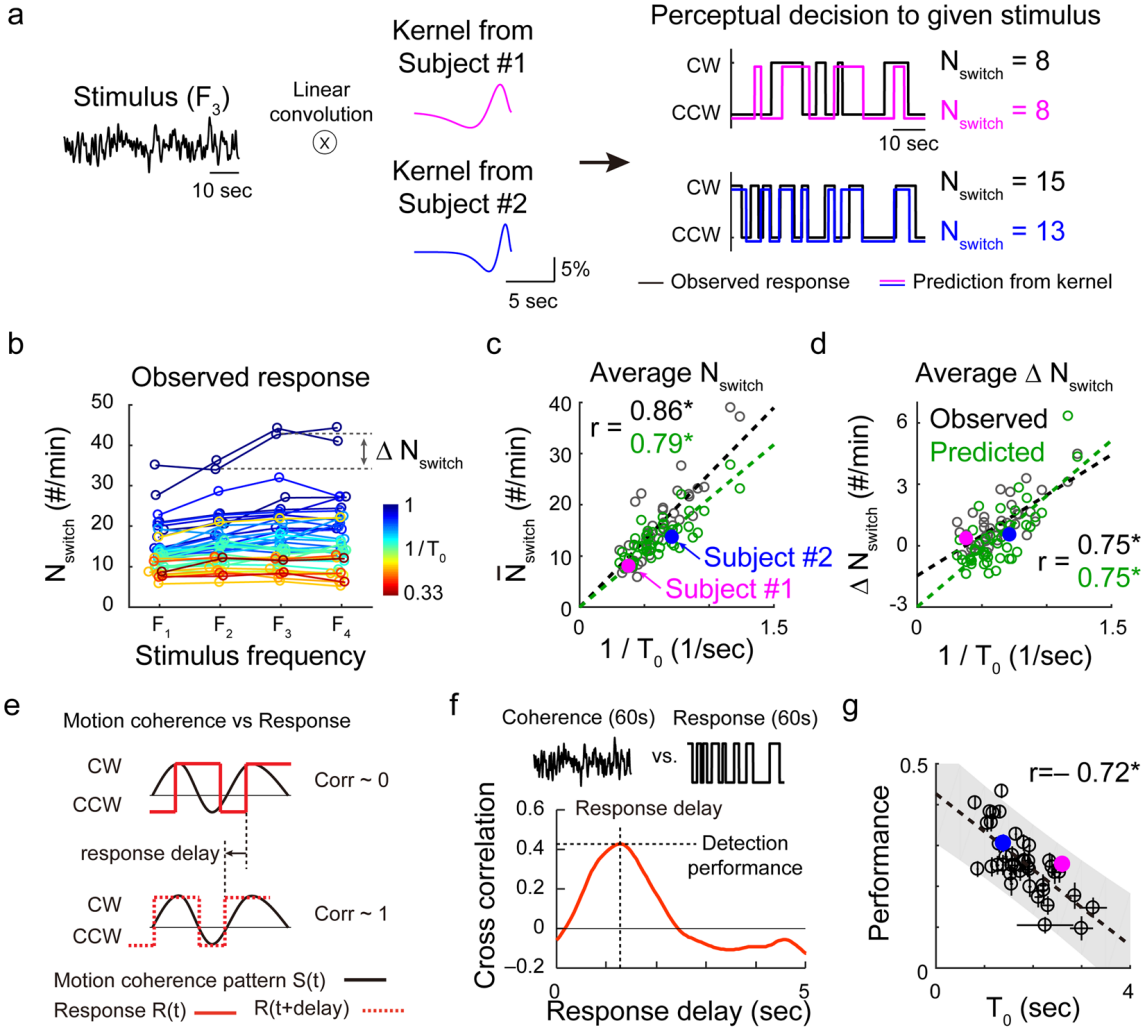
Observed sensory integration kernel predicts subject’s perceptual responses. (**a**) Prediction of perceptual responses with observed kernels. Motion coherence pattern was linearly convoluted with the observed kernel and discretized (see Methods for details). The number of perceptual switches, N_switch_, was counted from the estimated and observed response pattern. This prediction matched the observed responses for a given stimulus well (see Supplementary Fig. S6 for details). (**b**) N_switch_ and ΔN_switch_ of subject responses were observed to compare with the prediction from the kernel. Each color represents data from different subjects of various T_0_. (**c**,**d**) Average N_switch_ was inversely related to T_0_ in both the model (kernel) prediction and observed data. ΔN_switch_ was also inversely related to T_0_ in the observed data, as predicted by the model. Colored filled circles show subject #1 and #2. (**e**) Motion detection performance and response delay. Ideally, the motion coherence pattern S(t) and response pattern R(t) must match a positive response delay. (**f**) Motion detection performance was defined as the maximum value of the cross-correlation between S(t) and R(t), and the response delay was defined as the time point when the cross-correlation curve reaches its maximum. (**g**) The T_0_ values of each subject were negatively correlated with the average performance (r = −0.72, p < 1.38 × 10^−7^, Pearson’s correlation coefficient, N = 42). Error bar denotes the standard deviation calculated from 100 sampling (see Method). Shaded area indicates the confidence interval of linear correlation. See Supplementary Fig. S7 for details.

Using the observed kernels, we tried to predict the subjects’ perceptual response to the stimulus in Fig. 1. From a linear convolution of the stimuli pattern and the observed kernel, we were able to successfully reproduce the perceptual response pattern and, in particular, N_switch_, defined as the average number of perceptual switches within 60 seconds trial, in each subject (Fig. 2a, see Supplementary Fig. S6 for detailed illustration). Our model predicted that the N_switch_ value of the subject would be inversely related to the observed kernel size T_0_, and this was confirmed by our observed data (Fig. 2b,c, r = 0.86, p < 2.20 × 10^−13^, between the observed N_switch_ and 1/T_0_, r = 0.79, 3.36 × 10^−10^ between the predicted N_switch_ and 1/T_0_, Pearson’s correlation coefficient, N = 42). In addition, our model predicted that subjects with small T_0_ would have large increment of N_switch_ as stimulus frequency increases, while subjects with large T_0_ would have fewer changes in N_switch_ across different stimulus frequency conditions. We measured the ΔN_switch_ of each subject (Fig. 2b) and confirmed that ΔN_switch_ is inversely related to the observed kernel size T_0_, as our model predicted (Fig. 2d, r = 0.75, p < 8.77 × 10^−9^, between observed ΔN_switch_ and 1/T_0_, r = 0.75, p < 1.35 × 10^−8^ between predicted ΔN_switch_ and 1/T_0_, Pearson’s correlation coefficient, N = 42).

If the individual sensory integration kernel size determines the number of perceptual switching during the task, we may then assume that the motion detection performance and the response delay of each subject are also governed by the kernel size T_0_. For instance, an individual with small T_0_ may better detect the fast change of rotational direction than an individual with large T_0_. To validate this hypothesis, we defined the motion detection performance and the response delay using the cross-correlation between the stimulus and response patterns (Fig. 2e,f). Cross-correlation between motion coherence pattern S(t) and response pattern R(t) estimates the how two patterns are similar. Because the subjects’ responses must occur after a stimulus is given, we calculated the correlation value as we increased the delay of the response pattern (Fig. 2e), to find the value of the optimal performance and response delay. In this cross-correlation curve with time delay, motion detection performance was defined as the maximum amplitude of the curve, and response delay was defined as the time point at the maximum amplitude (Fig. 2f, see also Supplementary Fig. S7). We then tested whether kernel size T_0_, could predict the motion detection performance or the response delay of the individual, using 75% of the trials to extract the kernel and the other 25% of trials to measure the behavior (see details in Methods). As expected, the kernel size T_0_ of individual subjects was negatively correlated with motion detection performance (Fig. 2g) and the response delay was also strongly correlated with T_0_ (Supplementary Fig. S7e). Taken together, RTA could precisely measure the individual time course of perceptual decisions with intrinsic kernel size T_0_. We then expected that the observed subject-specific sensory integration kernel may be responsible for inter-individual variability in perceptual behavior and might enable us to predict individual performances under a given stimulus condition.

### Kernel-matched stimulus optimizes motion discrimination performance

**Figure 3:**
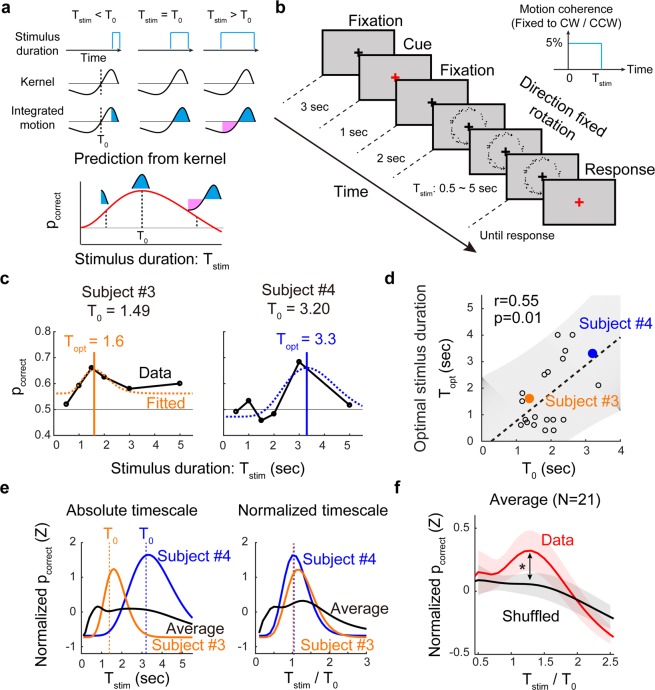
Kernel-size matched stimulus duration optimizes sensory perception. (**a**) Predicted perceptual response from the observed kernel. Our profile of the kernel predicts that the integrated motion evidence would be maximized when T_stim_ matches T_0_. If T_stim_ exceeds T_0_, the negative portion of the kernel contributes and the amount of integrated motion decreases. (**b**) Experimental design for finding an optimal value of stimulus duration. After fixation and cueing, the stimulus appears with a constant motion coherence (5%) with fixed rotational direction. The stimulus duration varied from 0.5~5 seconds. Subjects were instructed to report the direction of perceived motion at the last moment of the stimulus. (**c**) Optimal duration value at the peak p_correct_ varied across subjects. Two sample performance curves and their fitted value of optimal duration, T_opt,_ were shown. (**d**) Correlation between T_opt_ and T_0_. Optimal stimulus duration was strongly correlated with the observed kernel size T_0_ (r = 0.55, p = 0.011, Pearson’s correlation coefficient, N = 21). Colored filled circles show subject #3 and #4. Shaded area indicates the confidence interval of linear correlation. (**e**) In an absolute time scale, the p_correct_ curves from different subjects were noticeably different (left). However, in a timescale normalized by subjects’ T_0_ value, the curves appeared to have a similar pattern with a peak near 1 (right). (**f**) The averaged performance curves of normalized timescale increased as stimulus duration increased toward 1 (T_stim_ = T_opt_) and then gradually decreased. The maximum p_correct_ appeared at T_stim_/T_0_ = 1.2 and was significantly higher than the control, in which T_0_ values were shuffled (black) (Two-sided paired t-test, N = 21, p < 0.017, at T_stim_/T_0_ = 1.2). Shaded area denotes the standard error of the curve. See Supplementary Fig. S8 for details.

Based on the observations that subjects have various timescales of sensory integration, we predicted that the performance of subjects might be optimized by matching the stimulus pattern to the observed kernel profile. Our assumption was that if the evidence accumulation is governed by the observed kernel, integrated motion information would be maximized when the stimulus duration perfectly matches the size of the positive portion of kernel, T_0_. According to the temporal profile of our observed decision kernel, when the stimulus duration is shorter than the kernel size, the integrated information will increase as the stimulus duration increases. On the other hand, when the stimulus duration exceeds T_0_, the sum of integrated information decreases because the negative portion of the kernel starts to contribute (Fig. 3a, top). Thus, the probability of a correct decision would be maximized when the stimulus duration matches T_0_, and would decrease when the stimulus duration exceeds T_0_ (Fig. 3a, bottom). To validate this hypothesis, we designed our next experiment to have random dots generate a motion with a fixed direction (clockwise or counter-clockwise). The motion coherence was set at a constant level (5%), but the motion duration varied from 0.5 to 5 seconds. Subjects were asked to observe the stimulus until the end of the movie and then to report the motion direction perceived at the last moment of the stimulation, while the subjects were unaware of the fact that stimulus has a fixed rotational direction (Fig. 3b). If our assumption is correct, the accuracy of the perceptual decision will be highest when the stimulus duration matches to subject’s own T_0_, and is not high enough when the stimulus duration is shorter or longer than subject’s T_0_. Our experimental results confirmed that the probability of correct response, p_correct_ did not simply increase as the stimulus duration increased, rather they showed a peak at a certain value of stimulus duration in more than half of the subjects (Fig. 3c, subjects 3 and 4). To avoid the possibility of a hazard rate effect on the task, we checked whether p_correct_ does not increase as the trial number increases, and if p_correct_ does not increase as the stimulus duration increases. We found that p_correct_ does not show increasing tendency as the trial number increases, and that the average p_correct_ of the whole population does not increase as the stimulus duration increases. In fact, we found that only three subjects out of 19 showed maximum p_correct_ for the longest stimulus duration (5 s). This suggests that there exists an optimal size of evidence accumulation for making the correct decision (see Supplementary Fig. S8).

To examine whether the optimal perception occurs when stimulus duration is matched to the intrinsic kernel size, we fit the p_correct_ curve to an alpha function that can describe both increment and decrement of the p_correct_ curve. Then we estimated T_opt_, the stimulus duration that induces the maximum p_correct_ in each subject and compared it with the individual kernel size, T_0_. As expected, subjects’ T_opt_ was strongly correlated to T_0_ (Fig. 3d, r = 0.55, p = 0.0105, N = 21, Pearson’s correlation coefficient). We observed that the value of T_opt_ varied across subjects, according to their kernel sizes. (Fig. 3e, left, orange and blue). As a result, when the stimulus duration was given as a single fixed value, each subject would show a noticeably different performance.

When we normalized the time axis of each subject’s performance curve with their intrinsic kernel size T_0_, the performance curves instead showed a similar trend, which increased toward 1 (T_stim_ = T_opt_) and gradually decreased after (Fig. 3e, right, Fig. 3f, see Supplementary Fig. S8 for details). As a result, in the normalized time scale, the population average showed a peak around 1 (Fig. 3f, red solid line), suggesting that most subjects showed the maximum p_correct_ when the stimulus duration matched their intrinsic sensory integration kernel size. Taken together, these results confirm that sensory integration in an individual is governed by the observed non-linear kernel profile and the performance of a perceptual task may also vary, depending on the difference between the kernel size and stimulus duration.

### Illusory motion perception and sensory integration kernel

**Figure 4:**
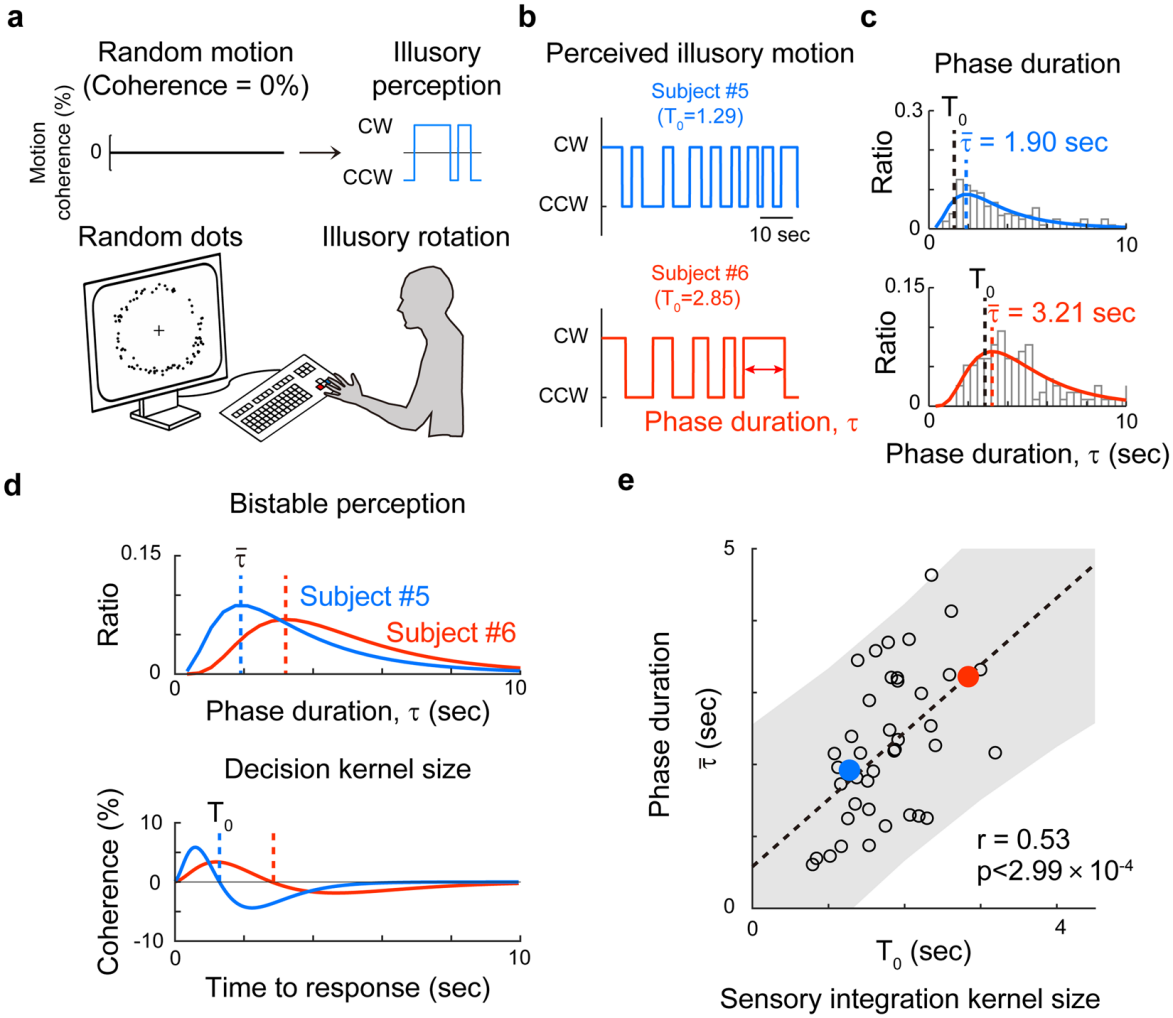
Sensory integration kernel predicts the periodic alternation in bistable perception (**a**) Random dot kinetics inducing illusory motion of bistable perception. Every dot is randomly distributed in each time frame, yielding no net motion. Most observers, however, perceived a rotating motion of the dots. (**b**,**c**) Sample responses from two subjects with a short (1.29 seconds, blue) and long (2.85 seconds, orange) T_0_ of integration kernel shown. In the bistable perception of illusory motion, subject 5 showed relatively faster alternation (top, blue) than subject 6 (bottom, orange) during 60 seconds of stimulation. The interval between two consecutive perceptual alternations was defined as the phase duration, τ. In each subject, the observed value of τ was fitted to a log-normal distribution and the peak value was denoted as $\bar{\tau }$. (**d**) The bistable phase duration $\bar{\tau }$ (top) and the size of the sensory integration kernel (bottom) of subject 5 and subject 6 were shown for comparison. (**e**) Correlation between the $\bar{\tau }$ and the size of the sensory integration kernel. A strong positive correlation was observed (r = 0.53, p < 2.99 × 10^−7^, N = 42, Pearson’s correlation coefficient). Shaded area indicates the confidence interval for linear correlation.

Thus far, our sensory integration kernel has been estimated from apparent motion signals. We further examined the notion that the observed intrinsic kernel may predict subjects’ behavior for illusory motion perception. Previous studies have shown that random dots scattered in an annulus induce an illusory rotational motion^26,27^ and that the perceived motion direction switches spontaneously between clockwise and counter-clockwise, showing a typical bistable perception dynamic^26,28,29^. We hypothesized that this periodic alternation in bistable perception might be also governed by the intrinsic kernel of subjects. To validate this hypothesis, we analyzed the response behavior in which subjects were asked to report the direction of the perceived motion from completely random dots (coherence, c = 0) were shown (Fig. 4a, see Movies S5). Consistent with previous studies, most subjects reported illusory rotational motion in this condition and the direction of perceived motion was periodically altered, spontaneously^26^. To quantify temporal features of this bistable perception, we measured the phase duration, τ, of illusory motion in one direction. Similar to a previous report^30^, we fit the measured τ values of a subject to a log-normal distribution and estimated the peak value $\bar{\tau }$, as a representation of individual dynamics of bistable perception.

The bistable phase duration, or $\bar{\tau }$, remained consistent within an individual, but varied across individuals. For example, Subject 5′s periodic alternation (Fig. 4b, top) appeared relatively faster than that of Subject 6 (Fig. 4b, bottom). The distribution of individual τ values were fit well to a log-normal distribution in most cases (Fig. 4c); thus the peak value of the distribution $\bar{\tau }$, was compared across subjects ($\bar{\tau }$ = 1.90 for Subject 5, 3.21 for Subject 6). The peak value, $\bar{\tau }$, varied greatly, from 0.5 to 5 seconds across subjects ($\bar{\tau }$ = 2.25 ± 1.10 seconds, mean ± S.D, see Supplementary Fig. S9). However, subjects who had a long intrinsic sensory integration time, T_0_, also tended to have slow switching dynamics with a large $\bar{\tau }$, while subjects who had a short intrinsic sensory integration interval tended to have fast switching dynamics with a small $\bar{\tau }.$ (Fig. 4d). As predicted, we observed a strong positive correlation between the values of $\bar{\tau }$ and T_0_, (Fig. 4e, r = 0.53, p = 2.99 × 10^−4^, Pearson correlation coefficient, N = 42). This strong correlation between the observed kernel size and the switching dynamics in bistable perception suggests that the observed intrinsic time of sensory integration may govern the perceptual response to illusory motions, as well as apparent motions.

## Discussion

Previous studies of motion perception have suggested that perceptual decisions arise through an accumulation of evidence, thus this process can be characterized by the drift-diffusion model^13,14^. In this bounded-evidence-accumulation model, the inter-individual variability in perceptual decisions is frequently explained by various conceptual parameters such as a decision boundary threshold, evidence accumulation rate, and choice bias^10,11^. The model can partially predict observed experimental results such as individual accuracy of perception. However, it still remains unclear what physical variables may indeed represent those decision parameters and if any of them are intrinsically consistent to characterize individual variance of subject behavior. Our result quantitatively describes the temporal profile of sensory integration for perceptual decisions, providing new insight into how decision variables should be implemented in the drift-diffusion model. Specifically, our result suggests that sensory integration is highly nonlinear in the temporal dimension, as observed in our kernel estimation, while the drift-diffusion model suggests that evidence accumulation (or the drift rate) is uniform over time. Moreover, our model can describe the decision variable as integrated sensory evidence in the drift-diffusion model, and the observed nonlinear integration kernel can then precisely describe how the drift rate changes nonlinearly, which is diverse across individuals. Thus, our finding of an intrinsic kernel suggests an alternative description of the drift-diffusion model and provides direct evidence that the intrinsic sensory integration interval is subject-specific and stimulus independent. Another notable issue here is that the amplitude of the integration kernel, A_pos_, varies with the stimulus condition (frequency), while the kernel size, T_0_, remains consistent (Supplementary Fig. S4). This suggests that subjects can modulate the total amount of motion integration depending on the stimulus condition while keeping the integration time constant during a response decision. Taken together, our finding of an intrinsic kernel suggests an alternative description of the drift-diffusion model and provides direct evidence of intrinsic sensory integration interval that is subject-specific and stimulus independent. Our results also suggest that the inter-individual variability in perceptual decisions may originate from this intrinsic sensory integration timescale and therefore may be considered a predictable trait.

One of the notable features of our sensory integration kernel is the existence of a negative portion of the profile. Similar to the current result, previous studies have also examined the extent to which present stimuli contribute to current decisions^20,31^, but only a positive contribution of the evidence was considered in such cases. In those reports, it was reported that stimuli presented early (1 s prior to the decision) affect the perceptual decision more strongly than those presented later. However, if we look at a small temporal window of the kernel of 1–2 s before the response, our observation showed trends identical those in the earlier findings (see Supplementary Fig. S10). Most likely, previous studies did not observe a negative portion because they only used a stimulus with a short duration and were thus unable to observe the integration process long before one second. The kernel observed in our study suggests that far before the decision (>1 s), the evidence does not always positively affect sensory integration. Then, one might ask by what mechanisms the negative portion of the decision kernel emerges? One possible candidate is the motion aftereffect. Because our motion stimulus is very long and consistent in time, it may generate a strong aftereffect that reverses the direction of the perceived rotational motion. Similar to previous findings that visual orientation perception can be negatively affected by the recent history of stimulus via tilt-aftereffect^32^, our motion perception can also be negatively affected by the motion aftereffect. If the motion aftereffect were strong enough to generate negative integration, our integration kernel must contain information on the diverse motion aftereffect profiles^33^ in individuals, which might be confirmed in the consequent study.

We were able to demonstrate that the observed sensory integration kernel can accurately predict diverse characteristics of perceptual behavior. In our first experiment, the number of perceived motion switching under the same stimulus conditions varied across the subjects (Fig. 2b) and this number was inversely related to the observed subject’s kernel size (Fig. 2c). Moreover, it was noticeable that subjects with shorter kernel size could detect the motion direction better than the subjects with the longer kernel size when the motion coherence of the stimulus fluctuated with different frequencies (Fig. 2g, Supplementary Fig. S7). Regardless of the stimulus frequency, subjects with the shorter kernel perceived the change of motion direction better than those with the longer kernel, potentially because a shorter integration kernel may induce less sampling error in integrating noisy coherent signals than a longer sampling kernel and therefore may be advantageous for encoding highly varying stimuli (see Supplementary Fig. S7b). Another noticeable result is the strong correlation between the response delay and the observed kernel size. In our observations, the response delay and the kernel size were almost identical; thus the response delay appeared very consistent within a subject and diverse across subjects, similar to the kernel profile (Supplementary Fig. S7e). In accordance with the previous observation of the relationship between decision time and motion discrimination accuracy^11^, this suggests that the timing of the subjects’ decision provides information about an individual’s decision process.

Contrary to anecdotal observations, we demonstrated that longer duration of constant motion stimulus did not enhance subject performance. Indeed, when the stimulus contains a constant motion with a fixed direction, a longer duration of stimulus would generate more information accumulated in the correct direction of the decision variable, therefore the drift-diffusion model predicts a higher correct ratio of the decision. In contrast, our observed sensory integration kernel has a highly non-linear structure with a positive peak and a negative overshoot thereafter. Thus, stimulus information provided within the size of the positive part of the kernel would enhance the performance, while a longer stimulus duration may induce negative drift and degrade the decision performance (Fig. 3a). As predicted by the observed kernel, our experiments showed that there exists an optimal stimulus duration for each subject and the subject’s performance became worse when the stimulus duration became longer than this length. Note that it is sub-optimal strategy^34,35^ to weigh any evidence negatively in the second experiment because subjects should not be affected by early evidence for optimal performance. However, our results show that most subjects negatively integrate early presented evidence when the stimulus duration is sufficiently long. This negative weighing may have been induced by motion aftereffects, indicating that the observed kernel profile reflects the complicated characteristics of sensory integration for motion perception instead of simple linear integration. Therefore, our second experiment suggests that sensory integration is not a simple linear accumulation, but can be predicted by the observed non-linear kernel within each subject T_0_ (Fig. 3e,f). This result raises an important issue; often, human psychophysics experiments are performed with fixed parameters of stimulus for all subjects and the responses are averaged across subjects to ignore inter-individual variation. Under these conditions, each subject will make a distinct decision behavior by their intrinsic kernels and the analysis could be misguided if we ignore the subject-specific traits. For example, if we simply average all the subject responses from a fixed timescale of stimuli, the averaged result may not show any clear trend (Fig. 3e, left). But, if we consider the subject-specific traits by kernel size so that the stimulus parameters were matched to the individual integration time, a common tendency of responses might be properly observed (Fig. 3e, right). This suggests that psychophysics experiments should be designed and performed carefully with a consideration of subject-specific differences.

Lastly, we showed that the observed kernel could predict the temporal features of bistable perception. The bistable perception in our third experiment is of a dynamic illusory motion, where subjects perceive a rotational motion of quasi-consistent duration from a totally random signal. For decades, it has been of interest to find the underlying mechanism of the bistable perception^36–39^, particularly on the origin of periodic alternation of perceived states. It has been reported that the bistable switching of frequencies from different types of stimuli is correlated in each subject, suggesting a common mechanism of bistable alternation^40–42^. We found a strong linear correlation between the phase duration of bistable perception and the sensory integration kernel size (Fig. 4e). Based on this, we argue that the origin of the subject-specific motion integration dynamics may be relevant to previous findings pertaining to bistable perception. First, it was reported that the gray matter volumes of the bilateral superior parietal lobes (SPL) were significantly correlated with the perceptual switching of a rotating structure-from-motion stimulus^17,18,43^. Specifically, individuals’ gray matter volumes of the anterior SPL (aSPL) were positively correlated with the phase duration and individuals’ gray matter volumes of the posterior SPL (pSPL) were negatively correlated with the phase duration. These outcomes suggest that the gray matter volumes of the superior posterior lobes of individuals determine the motion integration time. Second, it was also reported that the phase duration of bistable switching was significantly increased when lorazepam, a GABAa receptor agonist, was given to humans^44^. Similar to this result, computational models reported that inhibition can slow down the switching of bistable perception^44–48^. This suggests that the inhibition level of an individual brain may reflect the temporal scale of motion integration. Future studies should be conducted to confirm these notions.

In conclusion, we were able to verify an individual profile of sensory integration kernel from our controlled random dot stimulus and showed that human perceptual behaviors are governed by this kernel. The size of the kernel predicted an optimal stimulus duration for correct perceptual decision and the temporal characteristics of response under bistable conditions. Overall, our findings suggest that perceptual decisions arise in the intrinsic timescale of the sensory integration process.

## Methods

### Participants

Forty-five subjects (23 females, 22 males, ranging in ages from 20–29 years, with normal or corrected-to-normal vision) were enrolled in this study. All experimental procedures were approved by the Institutional Review Board (IRB) of KAIST (KH2017-05) and all procedures were carried out in accordance with approved guidelines. Written informed consent was obtained from all subjects.

### Display and visual stimulus

Visual stimuli were presented on an LCD monitor screen (DELL U3014, 29.8 inches, 2560 × 1600, 60 Hz temporal resolution) for all experiments. Subjects were positioned 160 cm away from the monitor and were asked to report their perception of the stimulus using buttons on the keyboard. At each frame of the stimulus, black dots were distributed in a circular annulus. The inner and outer radii of the annulus were at a 3.5 degree and 5 degree visual angle, respectively, from the center of the screen. The individual dots were 5 minute of solid angle in diameter and the dot density was set to 5 dots/deg^2^. The refresh rate of the visual stimuli was 20 Hz; thus, dots at each frame lasted for 50 ms and the dot locations were repositioned in the next frame. A black cross appeared at the center of the screen and each subject was asked to fix his or her eyes on the cross during the experiment. Stimulus conditions – including viewing distance, radii of the annulus, dot size, dot density, refresh rate, and the angle of rotation – were optimized based on the results from preliminary trials and previous references^26^ and was applied to all subjects. All visual stimuli were generated with MATLAB Psychtoolbox 3.0^49^.

### First experiment: Coherence-varying motion discrimination task

The 1^st^ experiment was comprised of five conditions. In one condition, the motion coherence level of the stimulus was set to 0 for a duration of 60 seconds (Fig. 4). In this condition, all of the dots in every frame were randomly located in the annulus and did not produce any global rotational motion. In the other four conditions, the motion coherence level of the stimulus, S(t), was set to fluctuate over time (Figs 1 and 2). In these conditions, S(t) was calculated from the following equation:

$${\rm{S}}({\rm{t}})={A}_{1}{\int }_{t=0}^{60}{C}_{0}(t)g(t-\tau )d\tau $$

where C_0_(t) is a random number from the normal distribution of *N*(0, 0.05) and g(t) is a Gaussian filter:

$$g({\rm{t}})=\frac{1}{{\sigma }_{filter}\sqrt{2\pi }}{e}^{\frac{-{t}^{2}}{2{{\sigma }_{filter}}^{2}}}$$

with four different σ_filter_ values of 100, 200, 400, and 800 ms. A_1_ is a constant to normalize the amplitude of S(t) (A_1_ = 5.4, 7.6, 10.75, or 15.20 for σ_filter_ values of 100, 200, 400, and 800 ms, respectively), which results in the average value of absolute coherence amplitude (e.g., average |S(t)|) becomes 8% under four different frequency conditions (see Supplementary Fig. S1). The sign of S(t) determined the rotation direction (clockwise for positive, counter-clockwise for negative values). At each frame, dots of S(t) were rotated by an angle θ_rotate_ = ±5° in the next frame. The detailed statistics of S(t) are shown in Fig. S1.

### Second experiment: Kernel-matched motion discrimination task

In the stimulus in the 2^nd^ experiment (Fig. 3), the black cross appears in the center of the screen for fixation. Aft0er 3 seconds, black cross changes the color to red for cueing the upcoming stimulus. After 1 second of cueing, the black cross appears for 2 seconds, and then the black dots in the annulus are plotted on the screen. The dots were set to have a fixed rotational direction, clockwise (CW) or counter-clockwise (CCW), which lasted for T_stim_. During T_stim_ (stimulus duration), the coherence level was fixed at 5%. Stimulus duration, T_stim_, was randomly chosen from the pool [0.5, 1, 1.5, 2, 3, 5] seconds (Fig. 3b). The sequence of stimulus duration conditions was randomized that subjects cannot predict the stimulus duration of the current trial.

### Behavior

In the first experiment (Figs 1, 2 and 4), subjects viewed rotating dots on the screen and were asked to report the direction of rotation by pressing the arrow keys on the keyboard whenever they perceived a change in the rotational direction of the dots (the right arrow key for clockwise rotation, the left arrow key for counter-clockwise rotation, and the down arrow key for mixed or ambiguous rotation). After the first direction report, subjects were asked to report the changes in direction via the corresponding key of a keyboard. Subjects were informed to press the down arrow key (mixed or ambiguous button) whenever the subject perceived no motion, strong motion in non-angular direction or both clockwise and counter-clockwise motion simultaneously. During the entire experiment, subjects rarely reported mixed/ambiguous rotation (less than 0.15% of time on average) and all of the subjects perceived rotational motion while watching^26,27^. Prior to data acquisition, subjects watched 30 s of random dots with no coherent motion (i.e., motion coherence level = 0 during stimulation), and before the main experiment, subjects performed one training session that contained three trials of 60 s to be familiar for the keyboard report. In the first experiment, each subject performed a total of 80 sequences of the trials: 64 trials (16 trials × 4 frequency conditions) of a coherence-varying motion condition and 16 trials of a random motion condition (S(t) = 0), with a random sequence of conditions.

In the second experiment, subjects were asked to fixate on the center of the screen and be aware of the upcoming stimulus when the red cross appeared. When dots appeared on the screen, subjects were asked to concentrate on the stimulus for the entire duration with no keyboard response. After visual stimulation ended, subjects were asked to report the rotational direction of the stimulus perceived at the last moment of the stimulus. Specifically, they were instructed to report the perceived direction for two possible cases; first, if they perceived no change in the motion direction through the stimulus duration, they simply reported the perceived direction, and second, if they perceived changes in the motion direction, they reported the very last direction perceived. To ensure that the subjects attended during the entire stimulus duration, we asked them to attend fully after the appearance of the red cross. Subjects were not informed that the given motion direction was fixed or that motion coherence was held constant. In the second experiment, each subject performed 50 perceptual decisions under six conditions of varying stimulus duration (300 total trials), with a randomly assigned sequence of the conditions.

Subjects performed two experimental sessions in a single day: the 1^st^ session consists of 20 trials in the 1^st^ experiment and the 2^nd^ session consists of 30 trials of the 2^nd^ experiment. Behavioral data was acquired over several days. In both the 1^st^ and 2^nd^ experiments, subjects did not receive any feedback during the experiments. Subjects were not informed about the experimental conditions (e.g., stimulus frequency of the 1^st^ experiment, constant motion coherence of the 2^nd^ experiment, etc.) and the objective of the experiments.

### Analysis

#### Extracting sensory integration kernel from coherence-varying motion discrimination task: Response-Triggered Average

To explore the subject-specific profile of the sensory integration kernel, the time course of the sensory integration for the perceptual decision was extracted from the 1^st^ experiment for each subject (Fig. 1). To extract a subject’s kernel, we first measured the time point at which the perceptual switch was reported, t_switch_. In a single frequency condition, F_*i*_ of motion coherence fluctuation, we extracted the motion coherence pattern 10 seconds prior to every j^th^ response of switching time, t_switch_ = _j_ and averaged these response-triggering motion coherence patterns as follows:

$${{\rm{RTA}}}_{{F}_{i}}=\sum _{switch=j}^{{N}_{switch}}\frac{sgn(switch)\,{S}_{{F}_{i}}({t}_{switch=j}-10\, \sim \,{t}_{switch=j})}{{N}_{switch}}$$

To obtain the average integration kernel of a subject, the RTAs from four different frequency conditions were averaged:

$${{\rm{RTA}}}_{{\rm{average}}}=\sum _{i=1}^{4}RT{A}_{{F}_{i}}/4$$

To minimize the possibility that the long and short RTAs came from the difference in switching numbers during the experiment, we generated a control response in which the responses were shuffled at random times, but with the same distribution of inter-response-interval. Then, the power of the kernel, P(t) = Σ (RTA(t)^2^) between the actual observed RTA and control RTA were compared (see Supplementary Fig. S3 for details).

After we obtained the RTA of each individual, we further investigated several factors that might generate bias on the shape of the RTA. First, we checked to see if the clockwise and counter-clockwise responses induced any differences in the RTA profile. We extracted an RTA using only CW or only CCW responses and compared two separate RTAs. Second, we checked to see if prior decisions could affect the RTA profile. To remove any contribution from prior decisions, we extracted the RTA using the condition that there was no other response between t = 0 (current decision) and t = 5, which is the RTA for a single decision. We compared the parameters of the kernel for this condition to the original kernel we observed. See the detailed results in Supplementary Fig. S5.

### Parameters to describe the shape of sensory integration kernel

Four parameters of the sensory integration kernel were defined. A_positive_ is defined as a positive amplitude of an individual RTA - sensory integration kernel, A_negative_ as a negative amplitude of the RTA, T_0_, as the first zero-coherence crossing of RTA, T_kernel_, as the timing when the negative RTA amplitude became less than 10% of the A_negative_ value. Correlations between each parameter were calculated and are reported in Supplementary Fig. S4.

### Motion energy analysis of the visual stimuli

While global rotational motion strength can be described by the designed motion coherence pattern, actual rotational motion strength presented to the subjects may vary locally in the annulus. To examine the net motion strength of the presented dot stimulus, we computed the motion energy of the stimulus in an angular dimension, following the previously published procedure^21^. Because the rotational motion is in an angular direction, we first summed the luminance value of each image frame radially, leaving the luminance value as a function of angular direction and time:

$$\bar{S}(\theta ,t)=\sum _{r=3.5^\circ }^{5^\circ }Stimulus\,Luminance(r,\theta ,t)$$

Then, to extract the local motion energy, a 24-degree pie window was chosen for calculating the local motion energy, and then was slid by 6 degrees with 18-degree overlap between adjacent spatial blocks. In total, we calculate the motion energy in 60 pie segments. The pie width (24 degrees) was chosen to match the size of the spatial filter we applied for motion energy analysis (approximately 2 visual degrees), and the moving window (6 degrees) was chosen to make enough segments (60 segments = 6°/360°), which could reduce the boundary artifact. Thus, the luminance value of the i^th^ pie segment was defined as:

$${\bar{S}}_{\angle {i}^{th}}(\theta ,t)=\bar{S}(6(i-1)^\circ  < \theta  < 24+6(i-1)^\circ ,t)$$

From the luminance value of each i^th^ pie segment (${\bar{S}}_{\angle {i}^{th}}(\theta ,\,t)$), the opponent motion energy was calculated by subtracting the counter-clockwise (CCW) rotational energy from the clockwise (CW) rotational energy:

$${\rm{ME}}{({\rm{t}})}_{\angle {i}^{th}}={\int }^{}CW\,energ{y}_{\angle {i}^{th}}(\theta ,t)d\theta -{\int }^{}CCW\,energ{y}_{\angle {i}^{th}}(\theta ,t)d\theta $$

The CW energy and CCW energy were calculated by squaring the linear convolution between the stimulus and spatiotemporal filter:

$$CW\,energ{y}_{\angle {i}^{th}}(\theta ,t)={({\bar{S}}_{\angle {i}^{th}}(\theta ,t)\ast C{W}_{1}(\theta ,t))}^{2}+{({\bar{S}}_{\angle {i}^{th}}(\theta ,t)\ast C{W}_{2}(\theta ,t))}^{2}$$

$$CCW\,energ{y}_{\angle {i}^{th}}(\theta ,t)={({\bar{S}}_{\angle {i}^{th}}(\theta ,t)\ast CC{W}_{1}(\theta ,t))}^{2}+{({\bar{S}}_{\angle {i}^{th}}(\theta ,t)\ast CC{W}_{2}(\theta ,t))}^{2}$$

where * denotes the linear convolution and $C{W}_{i}(\theta ,t)$ and $CC{W}_{i}(\theta ,t)$ is two pairs of spatiotemporal filters selective for each direction and defined as:

$$\begin{array}{rcl}{{\rm{CW}}}_{1}({\rm{\theta }},{\rm{t}}) & = & fast(t)O(\theta )+slow(t)E(\theta )\\ {{\rm{CW}}}_{2}(\theta ,t) & = & fast(t)E(\theta )-slow(t)O(\theta )\\ {{\rm{CCW}}}_{1}({\rm{\theta }},{\rm{t}}) & = & -fast(t)O(\theta )+slow(t)E(\theta )\\ {{\rm{CCW}}}_{2}(\theta ,t) & = & fast(t)E(\theta )+slow(t)O(\theta )\end{array}$$

Following the references, fast(t) and slow(t) denotes the temporal filter^19,21^:

$${\rm{fast}}({\rm{t}})={(kt)}^{6}{e}^{-kt}[\frac{1}{6!}-\frac{\beta {(kt)}^{2}}{(8)!}]$$

$${\rm{slow}}({\rm{t}})={(kt)}^{9}{e}^{-kt}[\frac{1}{9!}-\frac{\beta {(kt)}^{2}}{(11)!}]$$

and $O(\theta )$ and $\,E(\theta )$ are the spatial filter of odd and even Gabor function:

$${\rm{E}}({\rm{\theta }})=\,\cos (2\pi f\theta ){e}^{{(\theta /\sigma )}^{2}}$$

$${\rm{O}}({\rm{\theta }})=\,\sin (2\pi f\theta ){e}^{{(\theta /\sigma )}^{2}}$$

with parameters *β* = 0.9 and k = 60 for the temporal filter, and f = 0.5 cpd and σ = 0.6° for the spatial filter.

Having this motion energy pattern in each local spatial segment $({\rm{ME}}{({\rm{t}})}_{\angle {i}^{th}})$, we took three actions: (1) calculated the similarity between the motion coherence pattern and global motion energy, (2) compared the kernel obtained from the motion energy to the kernel obtained from the motion coherence pattern (Figs 1d and 3) compared the extracted kernel in the four quadrants (upper, lower, right, and left segments) to investigate possible spatial bias while integrating the motion. To compare the extracted kernel in the four quadrants, we fit the coefficient A_0_ ~ A_4_ using linear regression model:

$${{\rm{RTA}}}_{{\rm{average}}}({\rm{t}})={A}_{0}+{A}_{1}RT{A}_{upperME}(t)+{A}_{2}RT{A}_{rightME}(t)+{A}_{3}RT{A}_{lowerME}(t)+{A}_{4}RT{A}_{leftME}(t)$$

where RTA_ME_(t) is the response-triggered-average in each quadrant using motion energy, and RTA_average_(t) is the response-triggered-average using motion coherence level (sensory integration kernel). For details, see illustration and analysis results in Supplementary Fig. S2.

### Predicting the subject’s perceptual response with observed sensory integration kernel

To predict a subjects’ perceptual response with the observed individual kernel, the data sets were divided into two subsets: 75% of the trials were sampled to estimate individual kernels, RTA_subset_(t) and another 25% of the trials were used to measure behavioral parameters, the response pattern R(t) as a validation set. This random sampling of estimation and validation sets was repeated 100 times, and the profile of RTA_subset_(t) was used to predict the R(t) at each sampling. To predict a subjects’ response R(t) to a given stimulus S(t), we took a linear convolution of the motion coherence pattern S(t) with the individual motion integration kernel RTA_subset_:

$$L(t)={\int }^{}RT{A}_{subset}(\tau )S{(t-\tau )}_{{F}_{i}}d\tau $$

thus L(t) shows the linear response to the stimulus.

We predicted that the response switches when the integrated response L(t) exceeds the threshold value, L_th_ were as following:

$${R}_{predicted}(t)=\{\begin{array}{c}+1(CW)\,when\,\,L(t)\ge {L}_{th}\\ -1(CCW)\,when\,\,L(t)\le -\,{L}_{th}\\ R(t-1)\,when-{L}_{th} < L(t) < {L}_{th}\end{array}$$

and the threshold value L_th_ was calculated from the observed kernel as:

$${L}_{th}=\sum _{t=-10}^{0}RT{A}_{subset}{(t)}^{2}$$

As a result, predicted response pattern R_predicted_(t) can be obtained from RTA_subset_(t), and it was compared to observed response, R_observed_(t). To examine the goodness-of-prediction, the cross-correlation between the R_predicted_(t) and the R_observed_(t) was calculated (see Supplementary Fig. S6). High positive peak denotes good prediction of response pattern. As a control, the perceptual response was switched at random times, while maintaining the same inter-response-interval of the actual response.

### Estimating the number of perceptual switching with kernel size T_0_

To show that the number of perceptual switches is predictable from a subject’s sensory integration kernel, we counted subjects’ switch response (CW to CCW; CCW to CW) during each 60 s trial (Fig. 2a) under each of the four frequency conditions. Then, the average number of responses in each frequency condition – ${N}_{switch;{F}_{i}}$ – was obtained in each subject. To calculate the inverse relation between T_0_ and N_switch_, we fitted the relationship between the subjects’ average number of responses ${\bar{N}}_{switch}=\sum _{i=1}^{4}{N}_{switch;{F}_{i}}/4$ and the inverse of subjects’ kernel size 1/T_0_ to the formula ${\bar{N}}_{switch}=\frac{{\rm{C}}}{{{\rm{T}}}_{0}}$, where C is a subject-specific constant of response rate (units of count). The same fitting was applied to the predicted response R_predicted_(t) from the kernel (Fig. 2a). For example, observed population data estimated C as 26.0 and the predicted response data estimated C as 21.2 (Fig. 2c). Pearson’s correlation coefficient between the ${\bar{N}}_{switch}$ and 1/T_0_ was calculated to show significance. To show the trend of N_switch_ changes under four different frequency conditions, ${\rm{\Delta }}{N}_{switch}=\sum _{i=1}^{3}({N}_{switch;{F}_{i+1}}-{N}_{switch;{F}_{i}})/3$ were calculated in the observed data and the predicted response. Then, the relationship between the ΔN_switch_ and T_0_ was fitted into the formula ${{\rm{\Delta }}N}_{{\rm{switch}}}=\frac{{{\rm{C}}}_{1}}{{{\rm{T}}}_{0}}+{C}_{2}$ where C_1_ and C_2_ are the fitting parameters from population data (Fig. 2d). The observed data estimates (C_1_, C_2_) were (3.93 and −1.46) and the predicted response estimates were (5,42 and −2.99). Pearson’s correlation coefficient between the ${\rm{\Delta }}{N}_{switch}$ and 1/T_0_ was calculated to show significance.

### Predicting motion detection performance and response delay with kernel size T_0_

To examine the motion detection performance and response delay of a subject’s behavior in the 1^st^ experiment, the cross-correlation curve between the stimulus S(t) and the response R(t) pair was calculated (Fig. 2e). Here, S(t) is the motion coherence level at each frame and R(t) is perceived direction at each frame (+1 for clockwise rotation, −1 for counter-clockwise rotation, and 0 for mixed rotation). The normalized cross-correlation CC(t) between the S(t) and R(t) was calculated (Fig. 2e and Supplementary Fig. S7). Motion detection performance was defined as the maximum value of CC(t) at t = 0~5 seconds (since all of the subjects’ cross-correlation curve showed positive maximum value before 5 seconds) and response time was defined as the time lag at which CC(t) reaches a maximum value (see Fig. 2e and Supplementary Fig. S7 and ref.^26^).

### Second experiment: Perceptual response to a motion of different duration

In the experiment with a short visual stimulation (Fig. 3), the trial was counted as correct if the reported direction was matched the stimulus rotational direction. Then, the probability of correct response, p_correct_ was defined as a number of correct responses per total trial number. Because the p_correct_ of different stimulus duration shows does not simply elicit increment, the fitting function formula describing the relation between the correct ratio and stimulus duration should contain both increment and decrement as the stimulus duration increases. Thus, the fitting function was chosen as an alpha function:

$${{\rm{p}}}_{{\rm{correct}}}({T}_{stim})={C}_{1}{(\frac{{T}_{stim}}{\tau })}^{n}{e}^{-(n-1)\frac{{T}_{stim}}{\tau }}+{C}_{2}$$

where n describes the slope of the curve, τ describes the time constant, C_1_ and C_2_ determines the curve amplitude and the baseline p_correct_. We compared root mean square error (rmse) and the coefficient of determination using a linear function (${{\rm{p}}}_{{\rm{correct}}}({T}_{stim})={p}_{1}{T}_{stim}+{p}_{2})$, Weibull function (${{\rm{p}}}_{{\rm{correct}}}({T}_{stim})=1-0.5{{\rm{e}}}^{{(-{T}_{stim}/\alpha )}^{\beta }}+c)$ and the alpha function for fitting the p_correct_ curve over stimulus duration, which confirms that the alpha function can fit the p_correct_ curve better than the other two can. The fitting quality— coefficient of determination, R^2^—of the subjects’ p_correct_ curve to the alpha function was 0.56 ± 0.21, which is higher than the R^2^ of the linear function (0.22 ± 0.24) and Weibull function (−0.10 ± 0.96) (see examples in Fig. 3c, and in Supplementary Fig. S8).

In each fitted p_correct_ curve of the individual, the time point of the maximum p_correct_ – T_opt_ was estimated (Fig. 3c). The Pearson’s correlation value between T_opt_ and kernel size T_0_ was calculated to determine if motion integration is governed by the observed kernel (Fig. 3d). Note that T_opt_ is solely estimated from 2^nd^ experiment and T_0_ is solely estimated from the 1^st^ experiment.

Next, we investigated the general trend of each subject’s behavior to determine whether the average p_correct_ was maximized at T_0_ (see Supplementary Fig. S8). From the fitted p_correct_ curve, we Z-scored the p_correct_ and then rescaled the time axis T_stim_ with respect to the subject’s kernel size, T_0_. After we obtained the normalized p_correct_ curve in the time domain, we averaged all subject curves. As a control, we rescaled each subject curve with shuffled T_0_ of another subject. See Fig. 3e,f, and Supplementary Fig. S8 for details.

In such experiments with variable stimulus durations, the hazard rate may affect the subject’s behavior. To avoid any unexpected effects of the hazard rate, we examined two possible scenarios. First, p_correct_ may increase as trial number increases in a single session. A single experimental session consisted of 30 trials of randomly assigned sequence of six different durations. Therefore, a subject may predict the stimulus duration later in the session. We measured the slope of p_correct_ as a function of the trial number, and the result did not show any meaningful trend of a correctness change (the slope was close to 0), rejecting the first scenario. Second, the correctness could increase over time in a single trial. When the stimulus duration exceeds a certain length, the subject can presumably predict the length of the stimulus because candidate durations longer than that would be limited. We investigated whether p_correct_ differed across the stimulus durations, and the results showed there were no significant differences, rejecting the second scenario, too. We summarize these results in Supplementary Fig. S8.

### Perceptual reponses to illusory motion in bistable condition

For the condition S(t) = 0 (Fig. 4), phase duration τ was defined as the time interval between each switch of the perceived state. For each 60-second trial, the initial 10 seconds of data were excluded for the adaptation stage and the lower 1% and upper 5% of τ data points were excluded. Measured phase durations were converted into a cumulative density function, then fit to a log-normal distribution as:

$${F}_{\tau }=\frac{1}{2}[1+{\rm{erf}}(\frac{\mathrm{ln}\,\tau -\mu }{\sigma \sqrt{2}})],$$

where

$${\rm{erf}}(x)=\frac{2}{\sqrt{\pi }}{\int }_{0}^{x}{e}^{-{t}^{2}}\,dt$$

The log-normal distribution is a logarithm form of the normal distribution; thus, the peak of the τ distribution is analogous to the mean of the normal distribution. Therefore, $\bar{\tau }$ was used as the representative figure of perceptual switching distribution and $\bar{\tau }$ was then estimated from the fitted function as:

$$\bar{\tau }={e}^{\mu -{\sigma }^{2}}$$

Fitting was performed using the MATLAB function ‘NonlinearLeastSquares’.

### Statistical test

P-values and the type of statistical test used in the analysis are denoted in each figure caption and in the main text. We used a repeated-measure ANOVA to examine individual differences across the frequency conditions. Pearson’s correlation was used for the analysis of all linear correlations. We used a random shuffling method for comparison between the control and observed data, as described in the main text and figure legends. We calculated the ANOVA-Bayes Factor to determine the consistency of the T_0_ values across different frequency conditions (Fig. 1f) and across the local quadrants (Fig. S2) using the *BayesFactor* package of R.

### Data exclusion

Forty-five subjects participated in the 1st experiment. Data from three subjects were discarded from the analysis because the subject reported extremely small number of responses within 60 s trials (average response < 5 per 60 seconds trial). Total number of the subjects used in the further analysis is N = 42. This twenty-four subjects participated in the 2^nd^ experiment, who also participated in the 1^st^ experiment. Data from three subjects were discarded from the analysis with the same criteria of the 1^st^ experiment, leaving total N = 21.

## Supplementary information

Supplementary Information

Movie S1

Movie S2

Movie S3

Movie S4

Movie S5
