# Supplementary material for: Intrinsic timescales of sensory integration for motion perception
Source: Sci Rep. 2019 Mar 8;9:3993. doi: 10.1038/s41598-019-40649-9 (PMC6408463; doi:10.1038/s41598-019-40649-9)
Supplement: Supplementary file 1 — Supplementary Information [file 41598_2019_40649_MOESM1_ESM.pdf]

Supplementary material

Intrinsic timescales of sensory integration for motion perception

Woochul Choi[1,2] & Se-Bum Paik[1,2 *]

[1]Department of Bio and Brain Engineering, [2]Program of Brain and Cognitive Engineering, Korea Advanced Institute of Science and Technology, Daejeon 34141, Republic of Korea

*email: sbpaik@kaist.ac.kr

This PDF file includes:

Figs. S1 to S10

Captions for Movies S1 to S5

Other Supplementary Materials for this manuscript include the following:

Movies S1 to S5

Supplementary material

Supplementary Figure S1

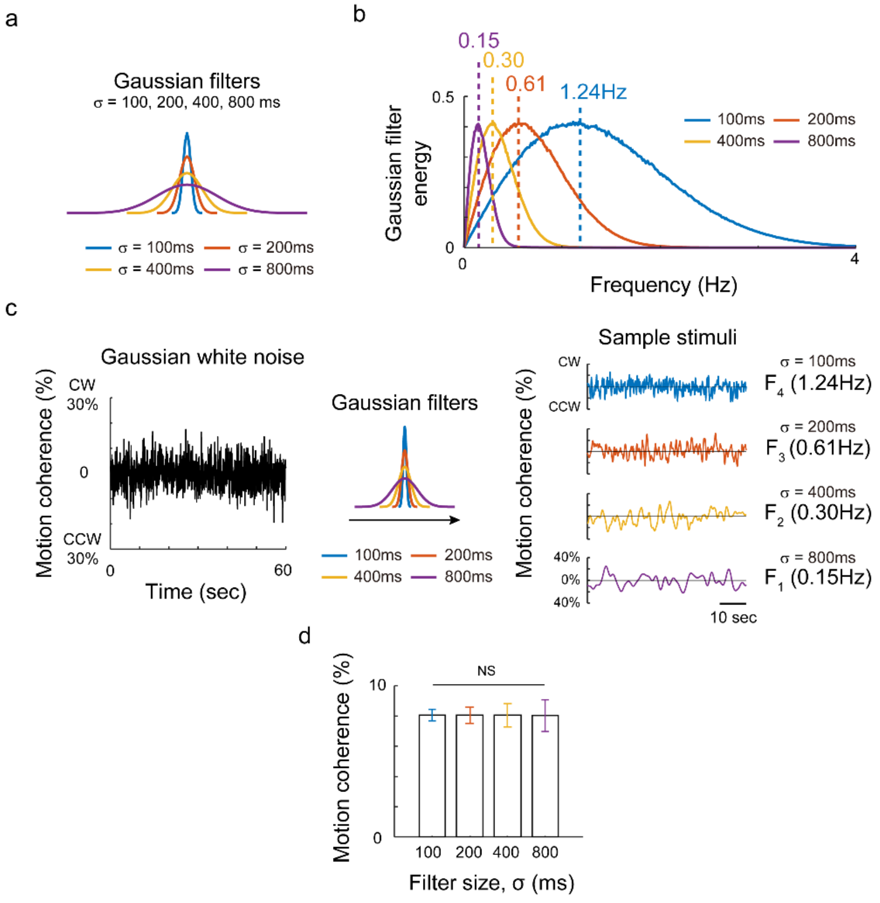

Fig. S1. Statistics of fluctuating motion pattern

(a) Preparation of visual motion stimulus. Four Gaussian filters were used to create a time-varying motion coherence of four different frequencies. Width of four Gaussian filters were 100, 200, 400 and 800ms (Blue, Orange, Yellow, Purple, respectively) (b) The energy of the Gaussian filters in frequency space. Each filter demonstrated a peak in the frequency-energy curve, which denotes the frequency for the highest energy. The peak appeared at 0.15, 0.30, 0.61, and 1.24 Hz when the stimulus was filtered with 800, 400, 200, and 100 ms Gaussian filters, respectively. (c) Gaussian random number was generated in every frame (left) and convoluted with a Gaussian filter, resulting in motion coherence pattern fluctuate with central frequency 0.15Hz (F_1), 0.30Hz (F_2), 0.61Hz (F_3) and 1.24 Hz(F_4). (d) In these four conditions, the average coherence was normalized to have the same value (8%, N=1000 simulations, one-way ANOVA, $F(3, 3996) = 0.28$, $p=0.91$). Because the average motion strength was equivalent in all conditions, thus the four conditions had the same task difficulty on average.

Supplementary material

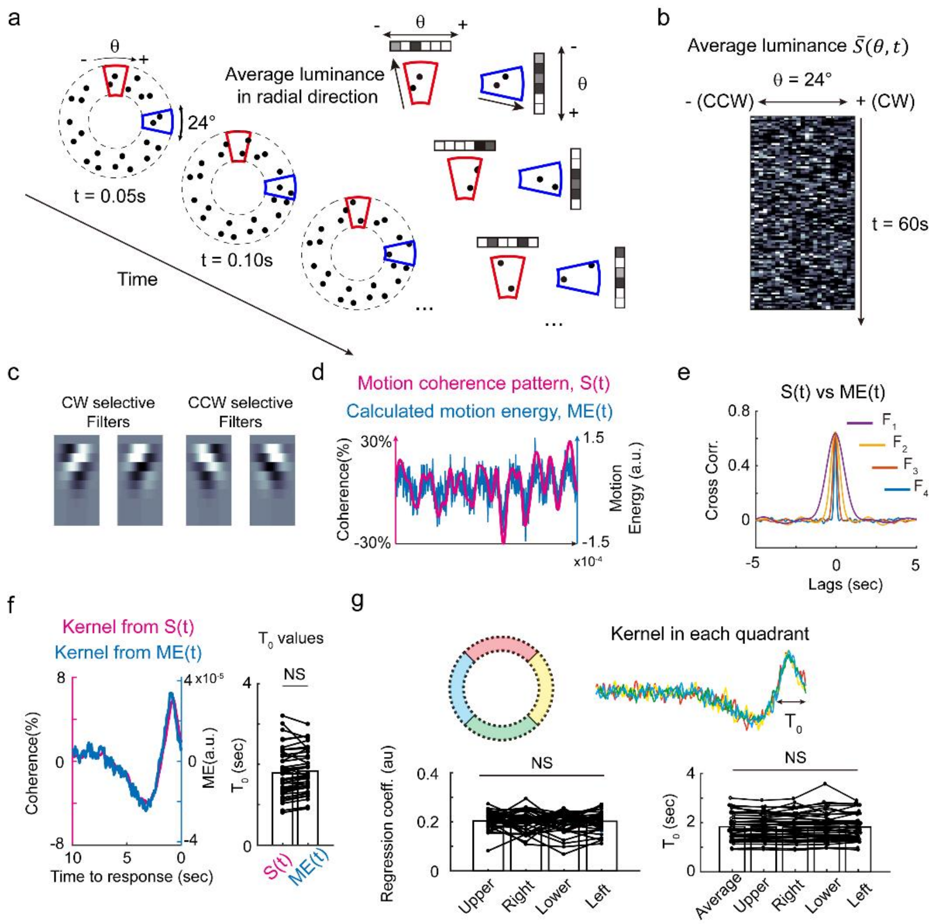

Fig. S2. Motion energy analysis

(a) Illustration of motion-energy-calculation procedure: A twenty-four-degree pie area was chosen as a local segment size (red and blue). The luminance value in each pie segment was averaged in the radial direction, resulting in average angular luminance, $\bar{S}(\theta, t)$. (b) Sample $\bar{S}(\theta, t)$ in θt dimension: Presented dot stimulus was converted to luminance value of each pie segment in θt dimension. (c) Sample spatiotemporal filters selective to clockwise and counter-clockwise direction: To estimate rotational motions, two pairs of spatiotemporal filters were implemented and were convoluted with $\bar{S}(\theta, t)$. (d) Sample motion coherence pattern and the global motion energy: Average motion energy fairly well follows the designed motion coherence pattern. (e) In each frequency condition, a high cross-correlation between the designed sample-motion-coherence pattern and the global motion energy confirms that the motion coherence pattern is well embedded in the presented dot stimuli. (f) A kernel extracted from motion energy: Similar to Fig. 1d, an individual kernel was calculated from temporally varying motion energy components. The T_0 values of two kernels were not significantly different (Two-sided t-test, $p < 0.07$, N=42) (g) Estimation of spatial bias in locally estimated kernels. To investigate if there is any spatial bias during the estimation of the kernel, we divided the stimulus space into four quadrants: upper, right, lower, and left (red, yellow, green and blue quadrants, respectively). We performed a regression analysis to check if each segment contributed differently to form the integration kernel. We found that the regression coefficients were not significantly different across the spatial locations (repeated-measures ANOVA, $F(3, 123) = 0.44$, $p = 0.72$, N=42, Bayes factor = 0.044). T_0 values were compared across the four quadrants and they were not significantly different across the spatial location and from their average (repeated-measures ANOVA, $F(4,160) = 1.66$, $p = 0.16$, N = 41, Bayes factor = 0.032).

Supplementary Figure S3

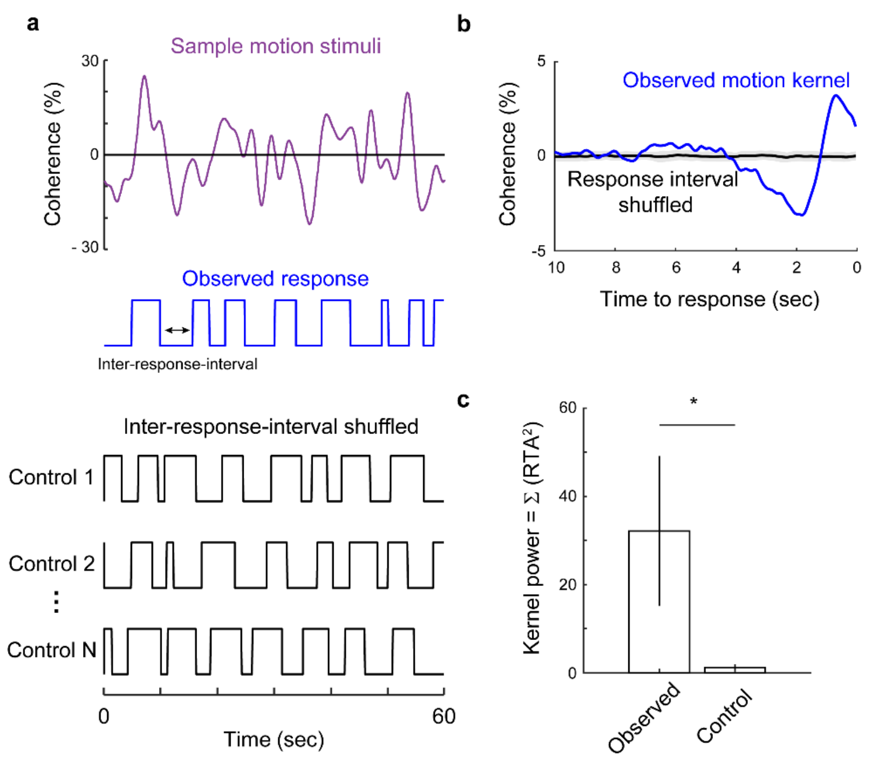

Fig. S3. Observed motion integration kernel and a control analysis

To reject the hypothesis that the observed kernel originated from the stimulus characteristics or from the individual variance of frequent/sporadic responses, we designed a control analysis. (a) With the stimulus used in the experiment (top, purple) and the observed response (middle, blue), we made a shuffled response maintaining the same inter-response-interval of the response (bottom, black). (b) We extracted the RTA from the observed response (blue) and control response (black). The observed kernel showed a significant peak in the curve, while no peaks were found in the control RTA kernel. Shaded area denotes the standard deviation of control RTA. (c) The control RTA from the same number of responses did not show a meaningful structure. The kernel power, defined as the sum of the squared RTA, was significantly higher in the observed RTA ($p < 4.15\times10^{-15}$, two-sided paired t-test, N= 42) than in the control.

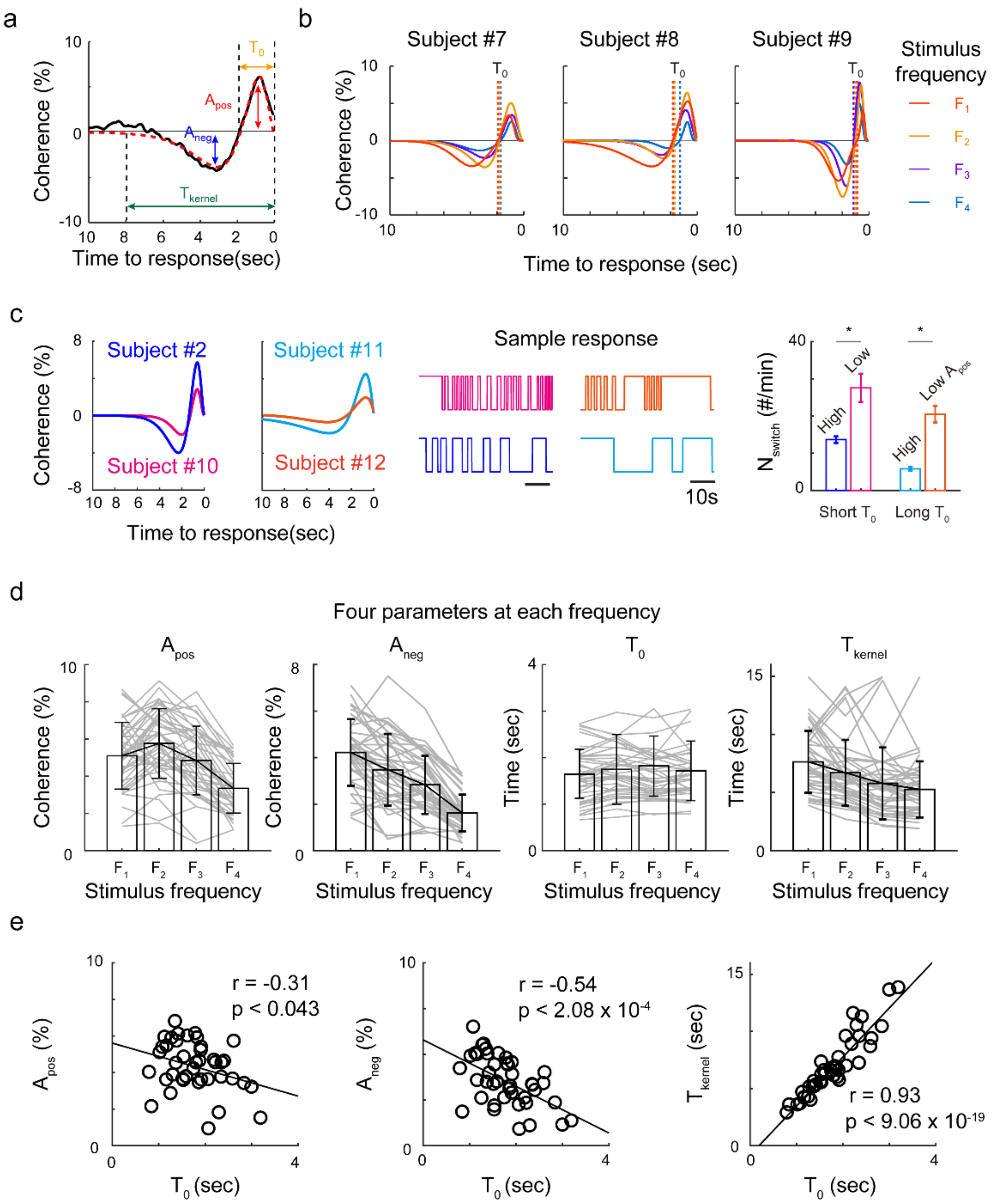

Fig. S4. Structural parameters of the sensory integration kernels

(a) To describe the sensory integration profile in detail, four parameters of the sensory integration kernel were examined: A_{pos}, A_{neg}, T_0, and T_{kernel}. (b) Sample kernels observed in three subjects under four conditions of stimulus frequency: T_0, the zero-crossing point of the fitted kernel under four conditions was shown as dashed lines. (c) To observe the impact of A_{pos} on individual behaviors, we compared two pairs of subjects (Subjects #2 & #10, #11 & #12, plotted in blue, magenta, cyan, and orange), whose T_0 values are very close to each other (#2 = 1.40 sec, #10 =1.27 sec, and #11 = 2.23 sec, #12 = 2.31 sec) but whose A_{pos} values vary greatly (#2 = 5.70%, #10 = 2.84% and #11 = 4.53%, #12 = 1.79%). The average N_{switch} values for each of the subjects for the frequency condition of F_3 are plotted. The N_{switch} of subject #2 was significantly smaller than that of subject #10 ($p < 2.71 \times 10^{-25}$), as was subject #11's compared to that of subject #12 ($p < 1.94 \times 10^{-13}$, N = 80 trials, Student's t-test), confirming that a subject with a lower A_{pos} switches their decision more rapidly than one with a high A_{pos}. (d) Four kernel parameters in each

frequency condition: A_{pos} appeared significantly different across the frequency conditions (repeated-measure ANOVA, $F(3,117) = 39.929$, $p < 7.64\times10^{-18}$), A_{neg} significantly decreased as the stimulus frequency increased ($F(3,117) = 103.15$, $p < 1.03\times10^{-32}$). T_0 was stable under all 4 conditions as shown in Fig. 1f. T_{kernel} slightly decreased as the stimulus fluctuation frequency increased ($F(3,117) = 22.787$, $p < 1.09\times10^{-11}$). It is important to note the significant differences in A_{pos} and A_{neg} across the stimulus conditions. This suggests that subjects modulate the total amount of evidence integrated depending on the stimulus condition while maintaining similar integration times. (e) Both individual A_{pos} and A_{neg} parameters were all negatively correlated with the individual T_0 value ($r = -0.31$, $p<0.043$ between A_{pos} and T_0, $r = -0.54$, $p < 2.08\times10^{-4}$ between A_{neg} and T_0, N= 42), and the T_{kernel} was positively correlated with the individual T_0 value ($r =0.93$, $p < 9.06\times10^{-19}$, N = 42, Pearson's correlation coefficient).

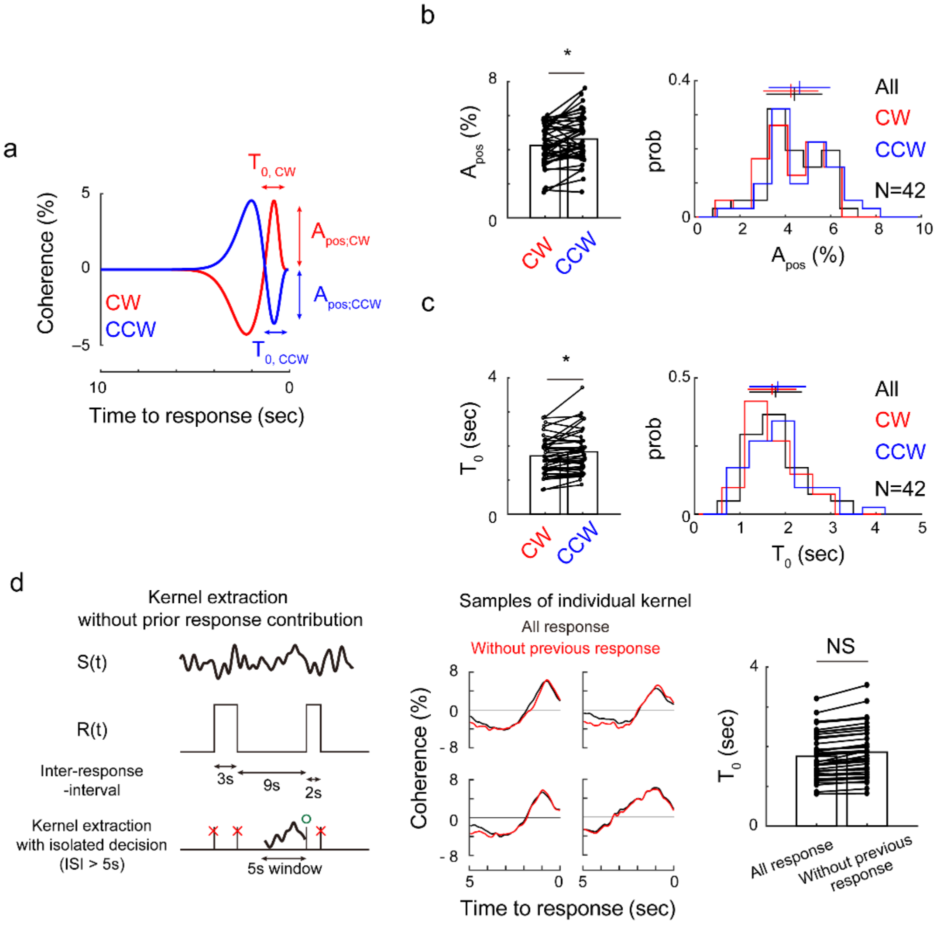

Fig. S5. Consistency of sensory integration kernel

(a) Sample CW and CCW kernels: To find any imbalance between the CW and CCW decisions, kernels were separately estimated from the CW or CCW responses. Observed kernels from the CW decision (red) and from the CCW decision (blue) showed a similar shape but opposite sign of coherence. (b) The amplitude A_{pos} of the CW and CCW kernels were different in individuals (Two-sided paired t-test, $p< 0.0038$, N=42), but the distribution of A_{pos} appeared not significantly different among the CW, CCW, and summed conditions. (c) The positive width T_0 of the CW and CCW kernels were also different in individuals (Two-sided paired t-test, $p<0.003$, N=42), but the distribution of T_0 appeared not significantly different among the CW, CCW, and summed conditions. (d) A kernel without a prior decision contribution:

Supplementary material

To reject the hypothesis that the sensory integration kernel is affected by the last-reported decision, the response-triggered-average stimulus was calculated only using an isolated decision, i.e. when no previous decision exists within a five-second window (left bottom). The sample kernels using all the responses (black) and kernel using only isolated response (red) is shown (middle). Note that kernel without previous response also have negative portion, which reveals previous decision report does not generate negative weighting of sensory evidence. The estimated T_0 value of the kernel without previous response was not significantly different from T_0 value of the original kernel (Two-sided paired t-test, $p=0.48$, $N=42$)

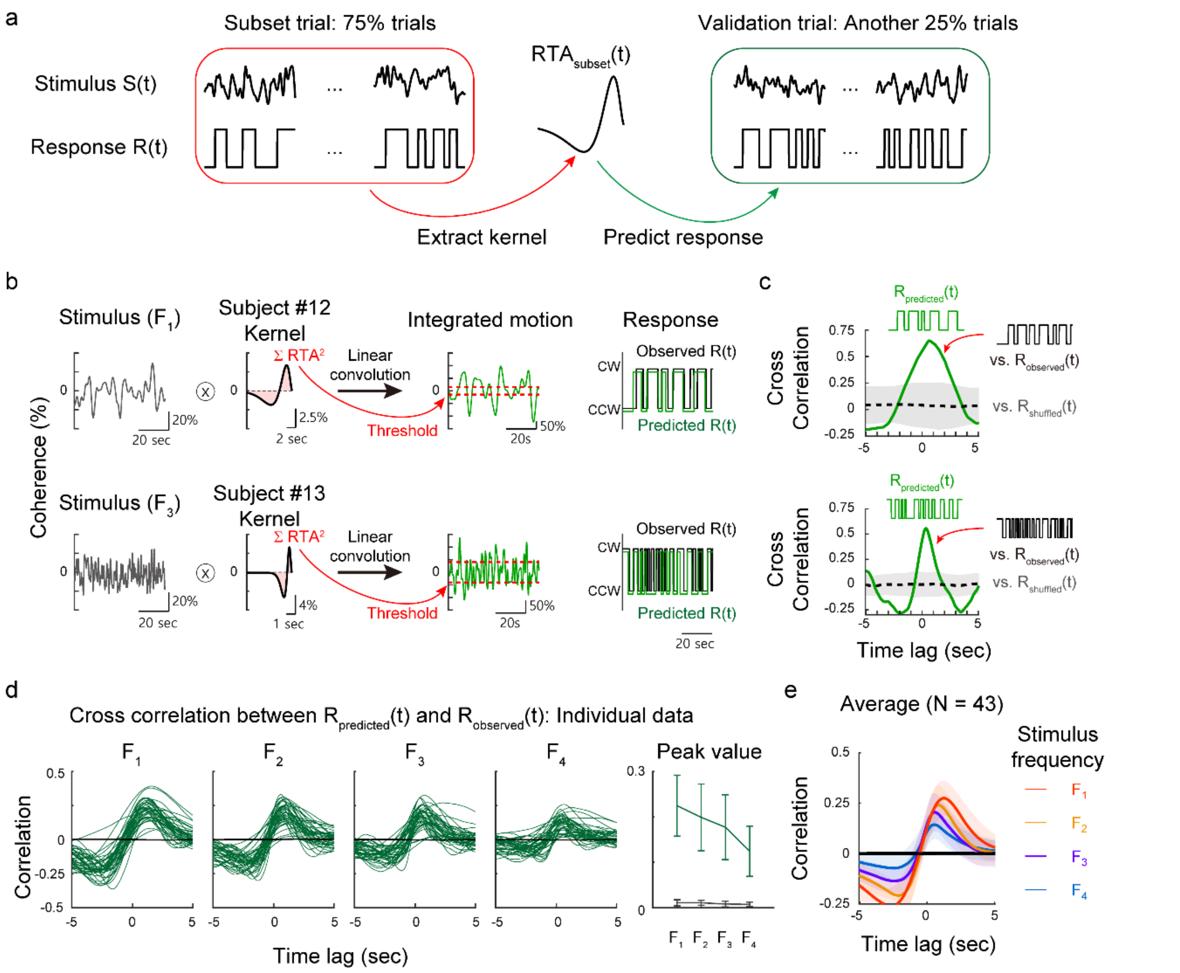

Fig. S6. Perceptual responses predicted from the linear convolution between stimulus and observed kernel

(a) To predict response behavior from individual kernels, data sets were divided into two subsets: 75% of the trials were sampled to estimate individual kernels, and the other 25% were used to examine response patterns. This sampling procedure was repeated 100 times, to estimate $RTA_{subset}(t)$. This $RTA_{subset}(t)$ was used to predict the response pattern, R(t) of validation trial. (b) Two sample individual response predictions are shown. First, a stimulus pattern was linearly convoluted with $RTA_{subset}(t)$ (left), and an integrated motion curve was obtained (middle). A threshold value was set from the square sum of the kernel (red dashed line, see Methods for details), and we assumed that a simulated response was switched if the linear response exceeded the threshold (see Methods for details). (c) To quantify the power of prediction, we calculated a cross-correlation between the observed response $R_{observed}(t)$ (black lines) and the predicted perceptual response $R_{predicted}(t)$ (green lines). The model successfully replicated the observed response, which was confirmed by the high correlation value (green lines). Correlations of the time-shuffled response data was also calculated as a control (black lines). Shaded areas denotes the standard deviation of the cross-correlation. (d) Cross-correlation of the model and observed data under four frequency conditions. Each line indicates the individual simulations. Significant peaks in the cross-correlation curve reveals that individual kernels can predict the response to any of the given stimuli. (e) Average cross-correlation of the predicted and observed response. Each line denotes the mean correlation curve from four stimulus conditions and the shaded area shows the standard deviation.

Supplementary Figure S7

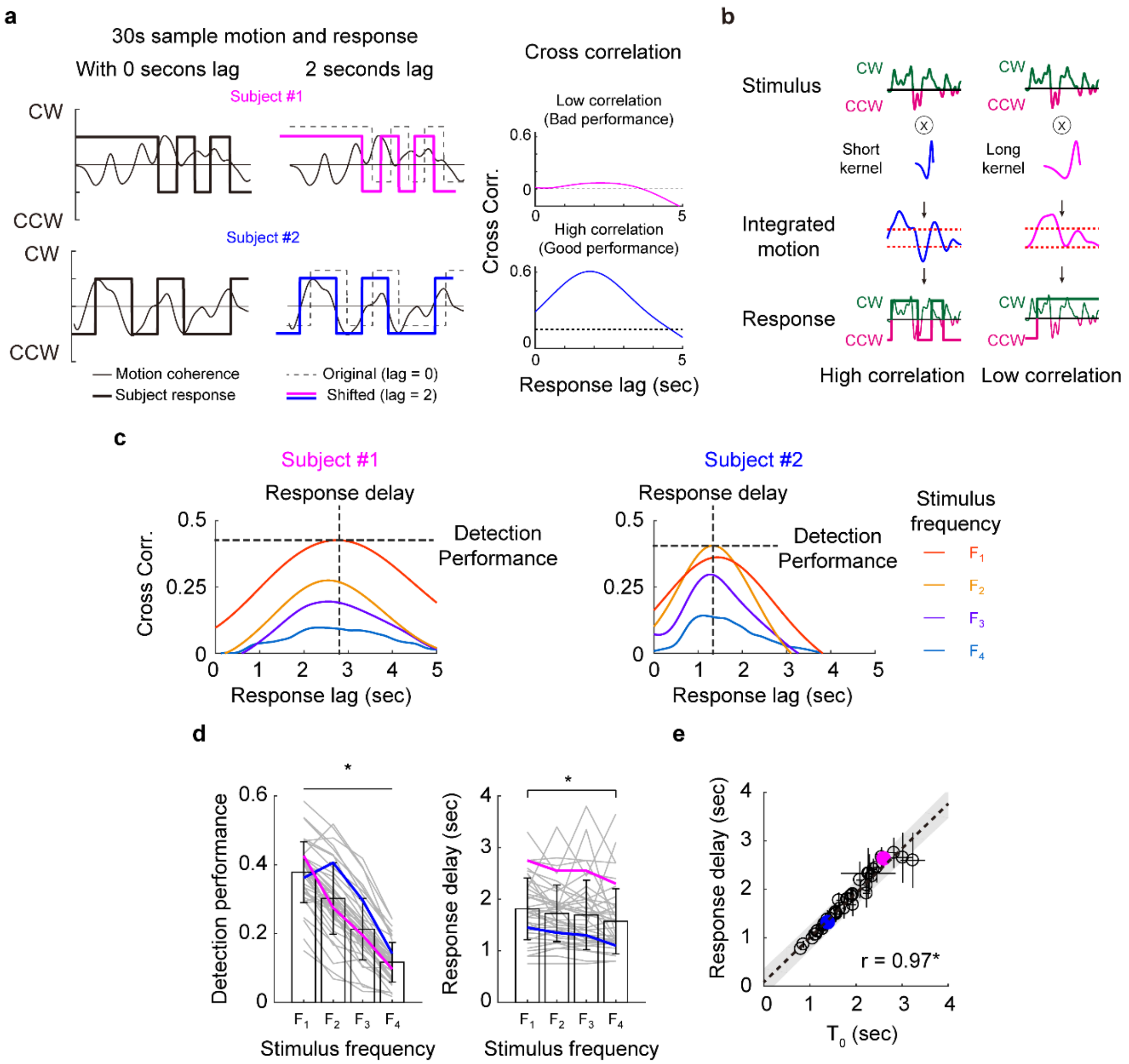

Fig. S7. Cross-correlation analysis of motion coherence pattern and perceptual response

(a) Two pairs of sample motion and response, and cross-correlation curves: Cross-correlation between motion coherence pattern and response was calculated with a positive delay. Subject #1's response poorly matched the given motion coherence pattern (top, pink), while Subject #2's response well matched the given motion coherence pattern with two seconds delay (bottom, blue). As a result, Subject #2's cross-correlation peak showed much higher amplitude (right bottom) than that of Subject #1 (right top). This peak value is defined as the accuracy of the first experiment task. (b) A possible mechanism that individuals with short kernel has higher accuracy than individuals with long kernel: Given a stimulus (top), each subject integrates the stimulus with their intrinsic kernel. As a result, subjects with a short kernel (blue) would integrate the stimulus with a short time window and the integrated motion would change quickly (middle). Thus, the response would show a high correlation to the given stimulus. However, subjects with a long kernel integrate the stimulus with large time window (magenta), so the integrated motion would moderately follow the stimulus pattern. Thus, this subject would not follow the fast stimulus and shows a weak

correlation between performance accuracy and T_0. (c) Two subjects' cross-correlation curves between the motion coherence pattern and response pattern were shown. The cross-correlation was measured (Fig. 2e) under the four stimulus conditions (F_1 ~ F_4). The maximum amplitude of the curve revealed the motion detection performance of the responses; the response delay was defined as the time point at which the correlation curve reaches the maximum value. (d) On average, accuracy decreased as the stimulus frequency increased ($p < 6.87\times10^{-63}$, $F(3, 123) = 394.17$, repeated measure ANOVA, N=42, average performance was 0.38 ± 0.09, 0.30 ± 0.10,0.21 ± 0.09, 0.12 ± 0.06 for F_1, F_2, F_3, F_4 respectively), and also the response delay slightly decrease as the stimulus frequency increased ($p < 0.003$, $F(3, 123) = 4.86$, repeated measure ANOVA, N=42, average response delay was 1.81 ± 0.60, 1.72 ± 0.55,1.70 ± 0.67, 1.57 ± 0.63 for F_1, F_2, F_3, F_4 respectively). (e) Strong positive correlation was found between T_0 and response delay of each individual ($r = 0.97$, $p < 5.84\times10^{-26}$, N=42, Pearson's correlation coefficient). Estimated response delay matched to the observed sensory integration kernel size T_0.

Supplementary Figure S8

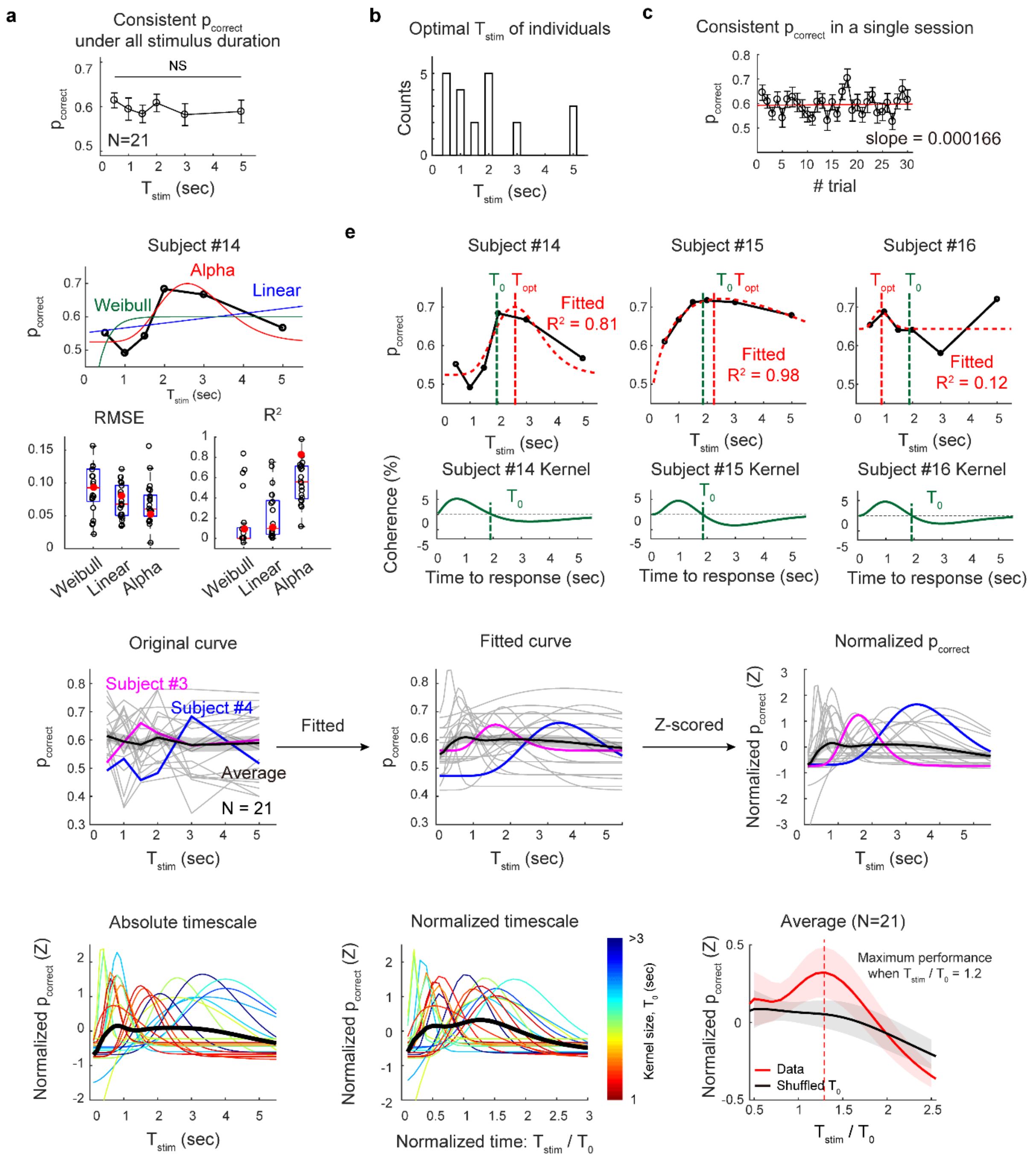

Fig. S8. Optimized stimulation enhances the perceptual performance

(a) Population average $p_{correct}$ across various stimulus duration conditions: $p_{correct}$ was not significantly different across all the duration conditions (repeated measure ANOVA). (b) Stimulus duration when $p_{correct}$ was maximum in each individual: Three out of 19 subjects showed maximum $p_{correct}$, at the longest stimulus duration (5s), while the other 16 subjects showed maximum $p_{correct}$ when the stimulus duration was shorter than the maximum. (c) To reject the hypothesis $p_{correct}$ increases as the experiment trials increases so that hazard rate influenced the perceptual decision, average $p_{correct}$ was measured as the trial increases in a

single session (Single session = 30 trials). The slope between $p_{correct}$ and trial number was close to 0 (slope of the curve = 0.00017), showing that the hazard rate effect barely affects $p_{correct}$. (d) A sample fitting result and the goodness of fit: The root-mean-squared-error (RMSE), and the coefficient of determination for each fitting function (linear, Weibull, and alpha function) are shown in the boxplot; each circle denotes the individual R^2. Red circle denotes the sample RMSE and R^2 of sample subject #14 $p_{correct}$ curve. (e) Sample correct ratio curves and integration kernels from three subjects: Two sample correct ratio curves of well-fitted subjects (14 and 15) and badly fitted subjects (16) are shown (top). T_{opt} was defined as the peak position of the curve (red dashed line). T_0 of each subject is shown (green dashed line). (f) Normalization of the correct ratio curves. The original curve (left) was fit to an alpha function (middle) and Z-scored (right). (g) Correct ratio curve in absolute and normalized timescales. The color denotes the value of T_0 in subjects. In a normalized timescale, the subjects had a similar trend. The population average showed maximum performance when T_{stim}/T_0 = 1.2 (right, red). As a control, the same correct ratio curve was normalized with shuffled T_0 of subjects (right, black). Shaded area denotes the standard error of the mean. A paired t-test at each time point showed that the grand average was significantly different from the control at T_{stim}/T_0 = 1.1 ~ 1.5 (Two-sided paired t-test, p = 0.037, 0.020, 0.017, 0.0023, 0.042, at T_{stim}/T_0 = 1.1, 1.2, 1.3, 1.4, 1.5, respectively, N=21).

Supplementary Figure S9

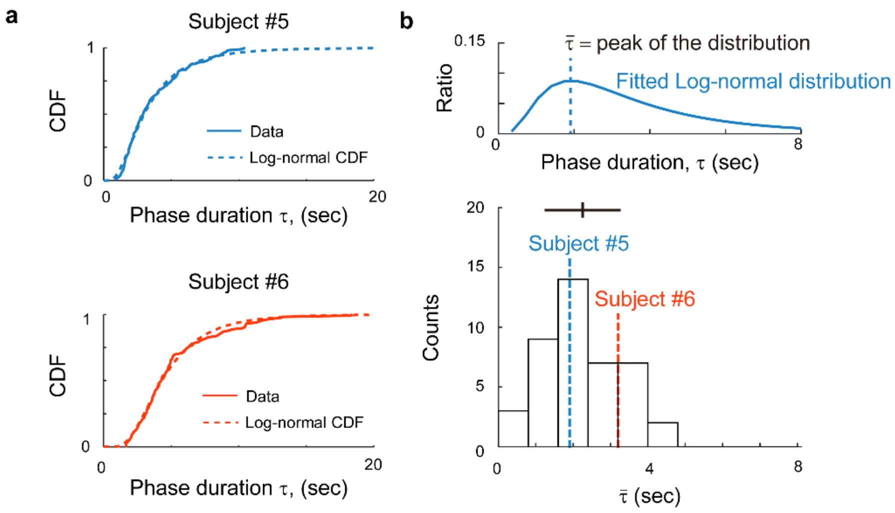

Fig. S9. Quasi-periodic switching behavior statistics under the random bistable condition

(a) The distribution of phase duration from two subjects. The τ distribution was first converted to a cumulative density function and then fit to a log-normal distribution. All subject τ distributions fit well to a log-normal distribution (Mean R^2 = 0.92, S.D. = 0.055), demonstrating that perceptual switching occurs in a quasi-periodic manner. (b) Histogram of individual τ statistics. The peak value, $\bar{\tau}$ varied from 0.5 to 8 seconds, while 90% of the subject's $\bar{\tau}$ values fell between 0.70 and 3.88 seconds. The population average and the standard deviation are shown with black solid lines.

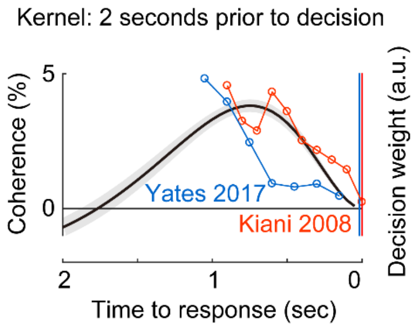

Fig. S10. Kernel compared to previous reference

Detailed kernel shape right before a response: In our observed kernel (black), a stimulus one second prior to the response affects the decision more strongly than that immediately before (<1 s) the response. The colored lines show previously reported decision weights that appeared to be similar with our observed kernel (orange – Kiani et al., 2008, blue – Yates et al., 2017).

Movie S1 – S5.

Movies S1 – S4 contain the 20-seconds sample stimulus video of F_1 ~ F_4 stimulus frequency (Fig. 1a). Movie S5 contains the 20-second sample stimulus video of random dot stimulation (Fig. 4a).
